# Genome-Wide Identification and Expression Pattern Analysis of the *DXS* Gene Family in *Eucommia ulmoides*

**DOI:** 10.3390/plants15182843

**Published:** 2026-09-17

**Authors:** Panfeng Liu, Xiujie Xue, Hongyan Du, Jiajia Zhang, Kunhao Xie, Gengxin Lv, Mengke Lian, Qingxin Du

**Affiliations:** 1 State Key Laboratory of Tree Genetics and Breeding, Key Laboratory of Forest-Derived Medicinal Resources, National Forestry and Grassland Administration, Research Institute of Non-Timber Forestry, Chinese Academy of Forestry, Zhengzhou 450003, China; pfengliu@caf.ac.cn (P.L.); 18369687185@163.com (X.X.); dhy515@caf.ac.cn (H.D.); jiajiazhang@caf.ac.cn (J.Z.); xiekunhao@163.com (K.X.); lianmengke2022@163.com (M.L.); 2 College of Horticulture, Ludong University, Yantai 264025, China; 3 Luoyang Academy of Agriculture and Forestry Sciences, Luoyang 471023, China; lv0623gx@126.com

**Keywords:** *Eucommia ulmoides*, *DXS* gene family, genome-wide identification, expression pattern, transient expression

## Abstract

1-deoxy-D-xylulose-5-phosphate synthase (DXS) is the first key enzyme in the methylerythritol phosphate (MEP) pathway of plant terpenoid biosynthesis, and it plays a vital role in *Eucommia ulmoides* terpenoid biosynthesis. In this study, bioinformatics methods were used to comprehensively identify and analyze the expression pattern of the *EuDXS* gene family, aiming to provide a basis for further functional study of *EuDXS* genes. A total of four *EuDXS* gene family members were identified and named *EuDXS1* to *EuDXS4*. The encoded proteins contained 625 to 713 amino acid residues, with molecular weight ranging from 67.98 kDa to 76.61 kDa. The theoretical isoelectric points varied from 6.79 to 8.69, aliphatic index was between 85.29 and 91.29. In silico subcellular localization prediction revealed that all EuDXS proteins were localized in chloroplasts. *EuDXS* gene members were categorized into three subfamilies, which were unevenly distributed on three chromosomes. The promoters of *EuDXS* genes contained various *cis-acting* elements related to stress response, phytohormone signaling, light response and growth regulation. Expression pattern analysis showed that *EuDXS* genes exhibited tissue-specific expression: *EuDXS1* was highly expressed in stem, leaf and fruit, *EuDXS2* was predominantly expressed in fruit. *EuDXS1* and *EuDXS2* exhibited high expression levels at the early developmental stage of fruits and leaves. In addition, *EuDXS* genes responded to salt and drought stress in varying degrees. Transient expression in tobacco revealed that *EuDXS1* and *EuDXS2* could increase carotenoid and total chlorophyll content. This study will provide important genetic resources for further exploration of *EuDXS* gene function and germplasm innovation in *E. ulmoides*.

## 1. Introduction

Terpenoids are among the most diverse and functionally extensive secondary metabolites in plants, playing a significant role in plant growth and development, stress defense, and pigment biosynthesis [1]. *Eucommia ulmoides* Oliver is a unique precious tree species endemic to China, whose fruit, bark and leaf can synthesize and accumulate abundant terpenoid secondary metabolites with great application value in pharmaceutical, industrial and other fields. Hence, investigations into the biosynthetic pathways and molecular regulatory mechanisms underlying terpenoid biosynthesis in *E. ulmoides* have attracted increasing research attention [2,3]. As an important high-molecular-weight polyterpenoid, the biosynthesis of *E. ulmoides* rubber mainly relies on the mevalonic acid (MVA) pathway and the methylerythritol phosphate (MEP) pathway [4]. 1-deoxy-D-xylulose-5-phosphate synthase (DXS) is the first rate-limiting enzyme in the MEP pathway. DXS catalyzes the reaction between pyruvate and glyceraldehyde-3-phosphate (G3P) to form 1-deoxyxylulose-5-phosphate (DXP). This process is the first step and acts as a crucial regulatory node in the MEP pathway [5].

*DXS* was first isolated from *Escherichia coli* [6], and subsequently identified in various plant species, including *Arabidopsis thaliana* [7], *Eriobotrya japonica* [8], *Solanum lycopersicum* [9] and *Lycium barbarum* [10]. It plays a crucial regulatory role in the biosynthesis of terpenoid and responses to abiotic stress. In loquat fruits, analysis of the *DXS* gene expression among different varieties during the color-change stage revealed that the content of carotenoids and cryptoxanthin was positively correlated with the expression level of *DXS* genes [8]. Overexpression of the *GmDXS1* gene in *Nicotiana tabacum* significantly increased the content of terpenoids (chlorophyll and carotenoids) [11]. Furthermore, overexpression of *DfDXS1* and *DfDXS2* derived from *Dryopteris fragrans* in tobacco significantly enhanced the drought and salt tolerance of transgenic plants [12]. The transcriptome sequencing of the fruits, leaves and barks revealed that the expression level of *EuDXS1* generally increased with the growth and development of *E. ulmoides* [13]. By constructing small RNA libraries from the leaves and fruits of *E. ulmoides*, it was predicted that *DXS* was the target of miR91 and the expression pattern of *DXS* is opposite to that of miR91. Therefore, the synthesis of *E. ulmoides* rubber could be promoted by utilizing the *DXS* gene through down-regulating the expression of miR91 in subsequent studies [14].

The plant *DXS* genes are encoded by multiple genes with distinct tissue-specific expression patterns and functional specificity [15]. To date, three types of *DXS* genes have been reported in plants: type I genes are housekeeping genes, with *AtDXS1* (*CLA1*) as a typical representative. Type II genes are mainly involved in plant secondary metabolism. For instance, *OsDXS3* has been confirmed to regulate plant defense responses and secondary metabolic pathways. Type III genes predominantly participate in the biosynthesis of low-abundance essential isoprenoids, such as plant hormones [16,17]. Although *DXS* genes have been characterized in various species, systematic research on the number, gene structure and functional regulation of the *EuDXS* gene family in *E. ulmoides* remains limited. Based on the genome of *E. ulmoides*, we comprehensively identified the members of the *EuDXS* gene family and conducted an extensive bioinformatics analysis of their physicochemical property, phylogenetic relationship, gene structures, conserved motifs, chromosome locations and promoter *cis-acting* elements. Combined with transcriptome data and qRT-PCR, the expression pattern of *EuDXS* family members in different tissues (stem, leaf, fruit and bark), under salt and drought stress treatment and at different developmental stages of fruits and leaves were analyzed. Furthermore, transient expression in tobacco was performed to investigate the regulatory roles of *EuDXS1* and *EuDXS2* involved in terpenoid biosynthesis. This study aims to provide a theoretical foundation for further exploration of the function of *EuDXS* genes.

## 2. Results

### 2.1. Identification and Physicochemical Property of EuDXS Genes

Four *EuDXS* genes were identified based on the *E. ulmoides* genome using HMMER and BLASTp, named *EuDXS1* to *EuDXS4* (Table 1). The physicochemical property analysis of EuDXS members revealed that EuDXS2 contained the largest number of amino acids (713), while EuDXS3 and EuDXS4 had the fewest amino acids (625). The molecular weight ranged from 67.98 kDa to 76.61 kDa, and the theoretical isoelectric point varied between 6.79 and 8.69. EuDXS2 was classified as an acidic protein with an isoelectric point below 7, whereas the others were alkaline proteins. The instability index ranged from 35.43 to 40.80. The aliphatic index was between 85.29 to 91.29, with EuDXS2 showing the lowest value. The average hydrophobic index was below zero, indicating that EuDXS members are hydrophilic proteins. In silico subcellular localization prediction demonstrated that all EuDXS proteins were distributed in chloroplasts.

The alpha-helix plays a crucial role in DNA-binding motifs, while random coil tends to form active and functional sites due to susceptibility to side-chain interactions. The secondary structure of EuDXS proteins consist of alpha-helices, extended strands and random coils (Table 2). The alpha-helix accounted for 36.96–40.03% with 231–277 amino acids. Random coil contained 304–353 amino acids, occupying 46.97–50.08%. The proportion order of secondary structures of EuDXS proteins was random coil, alpha-helix, extended strand.

### 2.2. Phylogenetic Relationship and Chromosomal Distribution of EuDXS Genes

**Figure 1:**
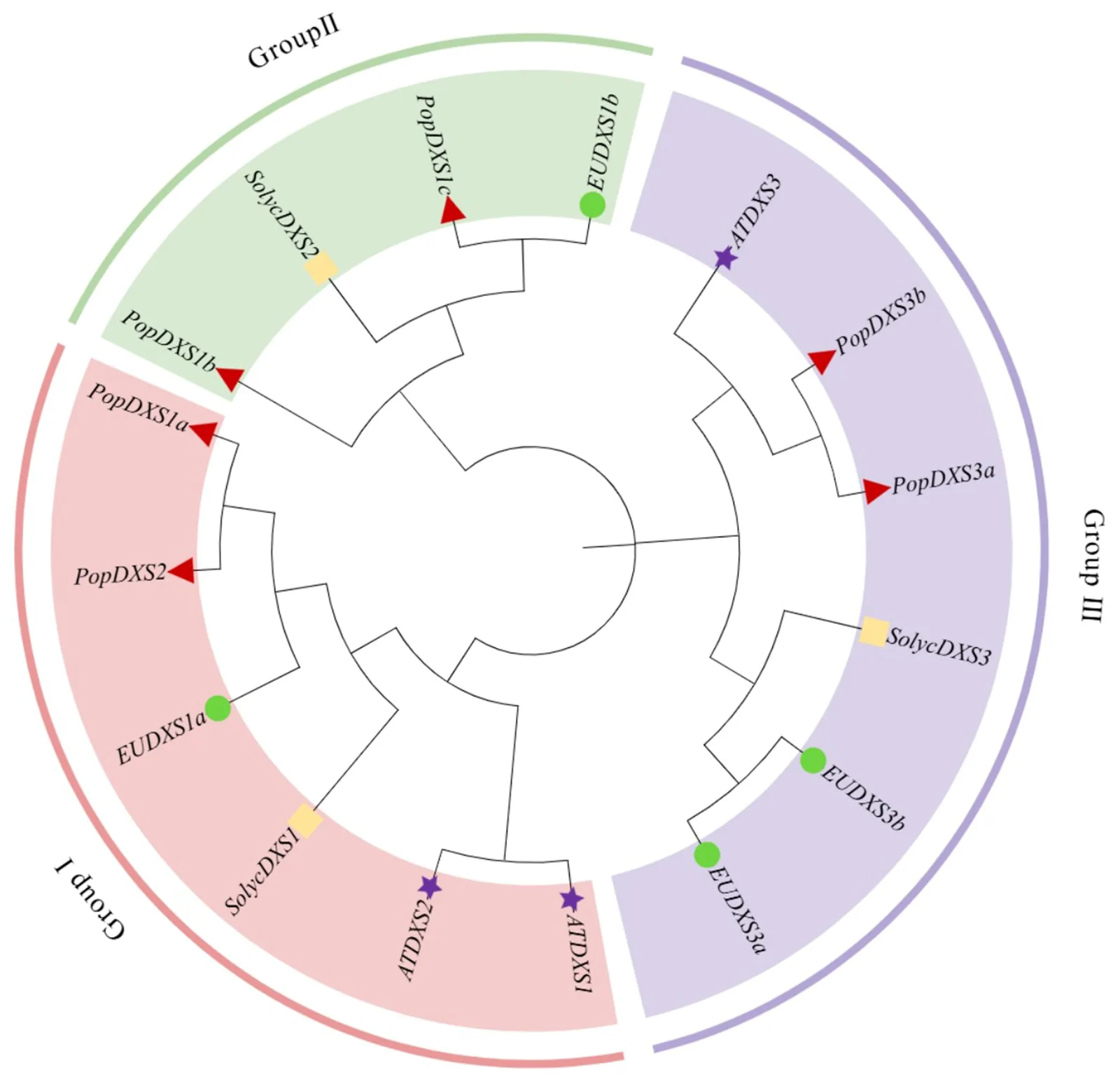
Phylogenetic tree of *EuDXS* Genes. Note: green circle represents *E. ulmoides*, yellow square represents *Solanum lycopersicum*, purple pentagram represents *Arabidopsis thaliana*, and red triangle represents ‘84K’ poplar.

To explore the phylogenetic relationship of *EuDXS* gene family members, a phylogenetic tree of *DXS* genes among four species was constructed, *E. ulmoides* (four genes for each), *Arabidopsis thaliana* (three genes for each), *Solanum lycopersicum* (three genes for each) and ‘84K’ poplar (six genes for each) (Figure 1). Sixteen DXS proteins were divided into three groups. Group I contained six members: EuDXS1, SolycDXS1, AtDXS1, AtDXS2, PopDXS1a and PopDXS2. Group II included four members: EuDXS2, SolycDXS2, PopDXS1b and PopDXS1c. Group III consisted of six members: EuDXS3, EuDXS4, SolycDXS3, AtDXS3, PopDXS3a and PopDXS3b. In addition, DXS proteins from four species were distributed in both Group I and Group III, indicating that the *DXS* genes in these two groups share high homology and close evolutionary relationships.

**Figure 2:**
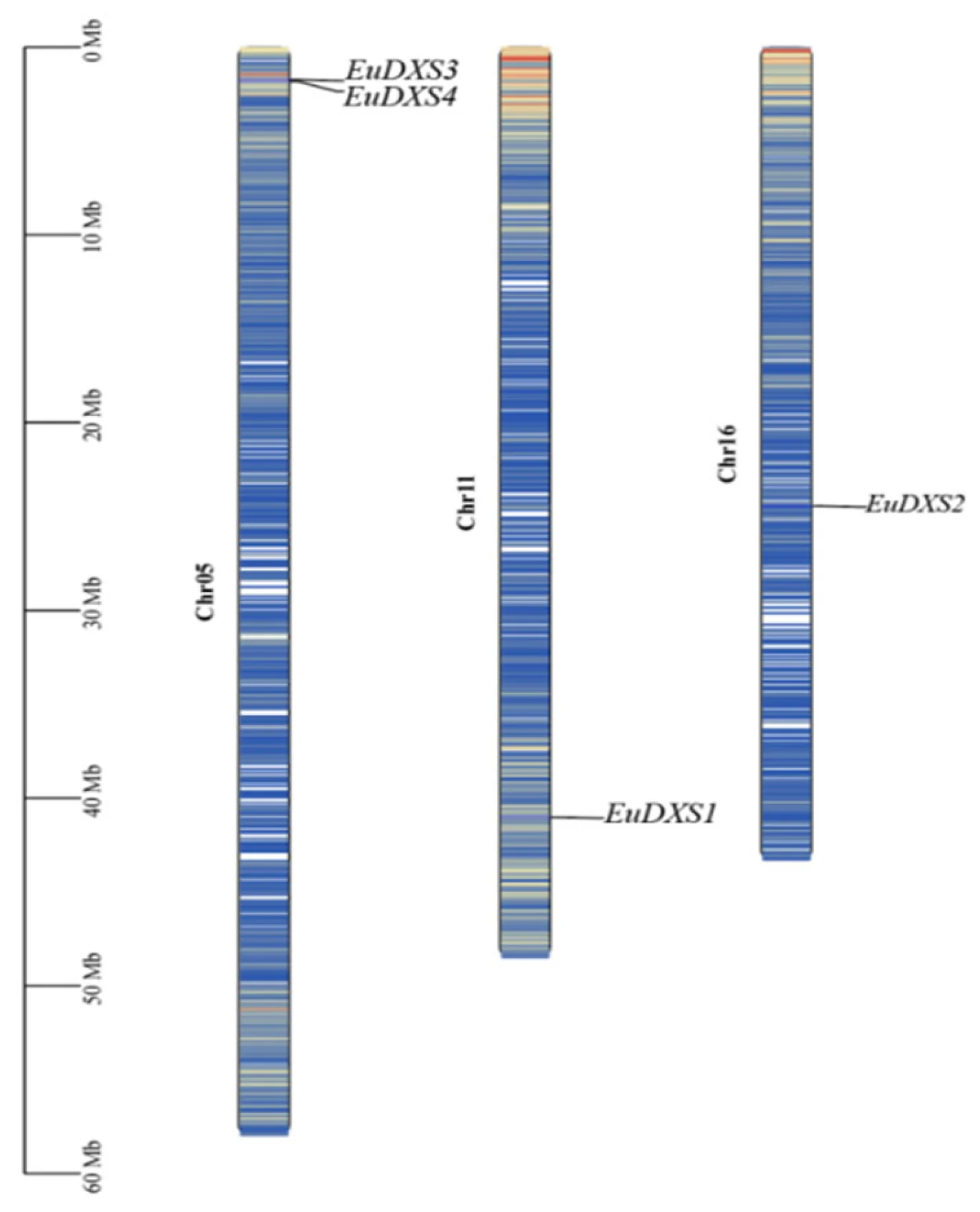
Chromosomal distribution of *EuDXS* genes.

Chromosomal localization analysis revealed that *EuDXS* genes were unevenly distributed across three chromosomes, namely Chr05, Chr11 and Chr16 (Figure 2). *EuDXS1* and *EuDXS2* were located on Chr11 and Chr16, respectively. *EuDXS3* and *EuDXS4* were adjacently located on Chr05.

### 2.3. Conserved Motifs and Gene Structure of EuDXS Genes

**Figure 3:**
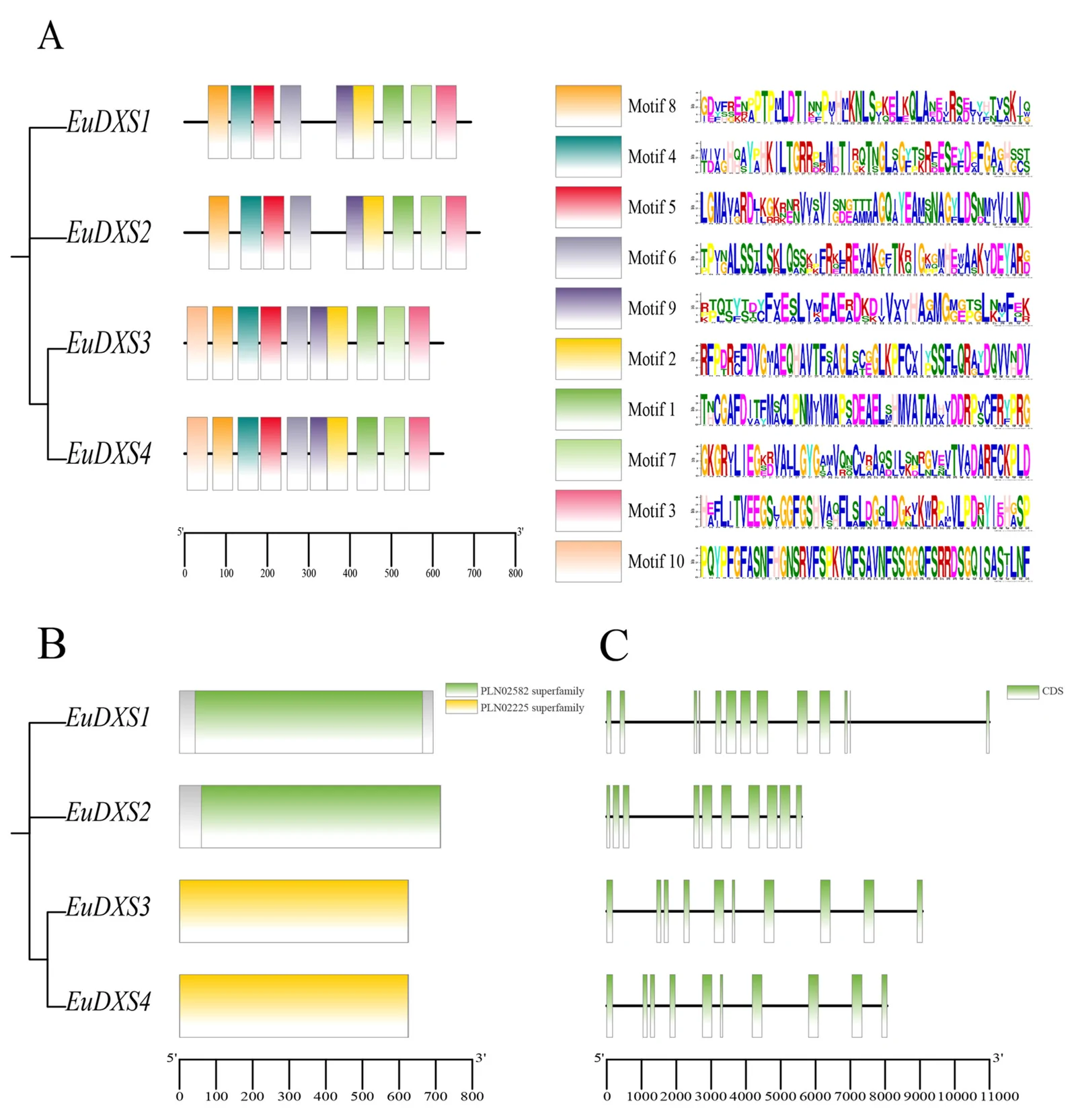
Motif composition and gene structure of *EuDXS* Genes. (**A**) Conserved motifs. (**B**) Protein domains. (**C**) Gene structure.

The motifs of *EuDXS* gene family members were analyzed by MEME-Suite (v5.5.1), and 10 distinct motifs were identified (Figure 3A). *EuDXS1* and *EuDXS2* contained nine motifs without motif10, exhibiting high similarity, while *EuDXS3* and *EuDXS4* contained all 10 motifs. Further analysis of domain structure of *EuDXS* genes revealed that both *EuDXS1* and *EuDXS2* contained the PLN02582 superfamily, while *EuDXS3* and *EuDXS4* possessed the PLN02225 superfamily, indicating that high structural similarity between *EuDXS1* and *EuDXS2*, as well as between *EuDXS3* and *EuDXS4* (Figure 3B). Variation in exon and intron numbers were observed among *EuDXS* family members (Figure 3C). *EuDXS1* had 13 exons and 12 introns, whereas the other three members all contained 10 exons and nine introns.

### 2.4. Cis-Acting Elements in Promoters of EuDXS Genes

**Figure 4:**
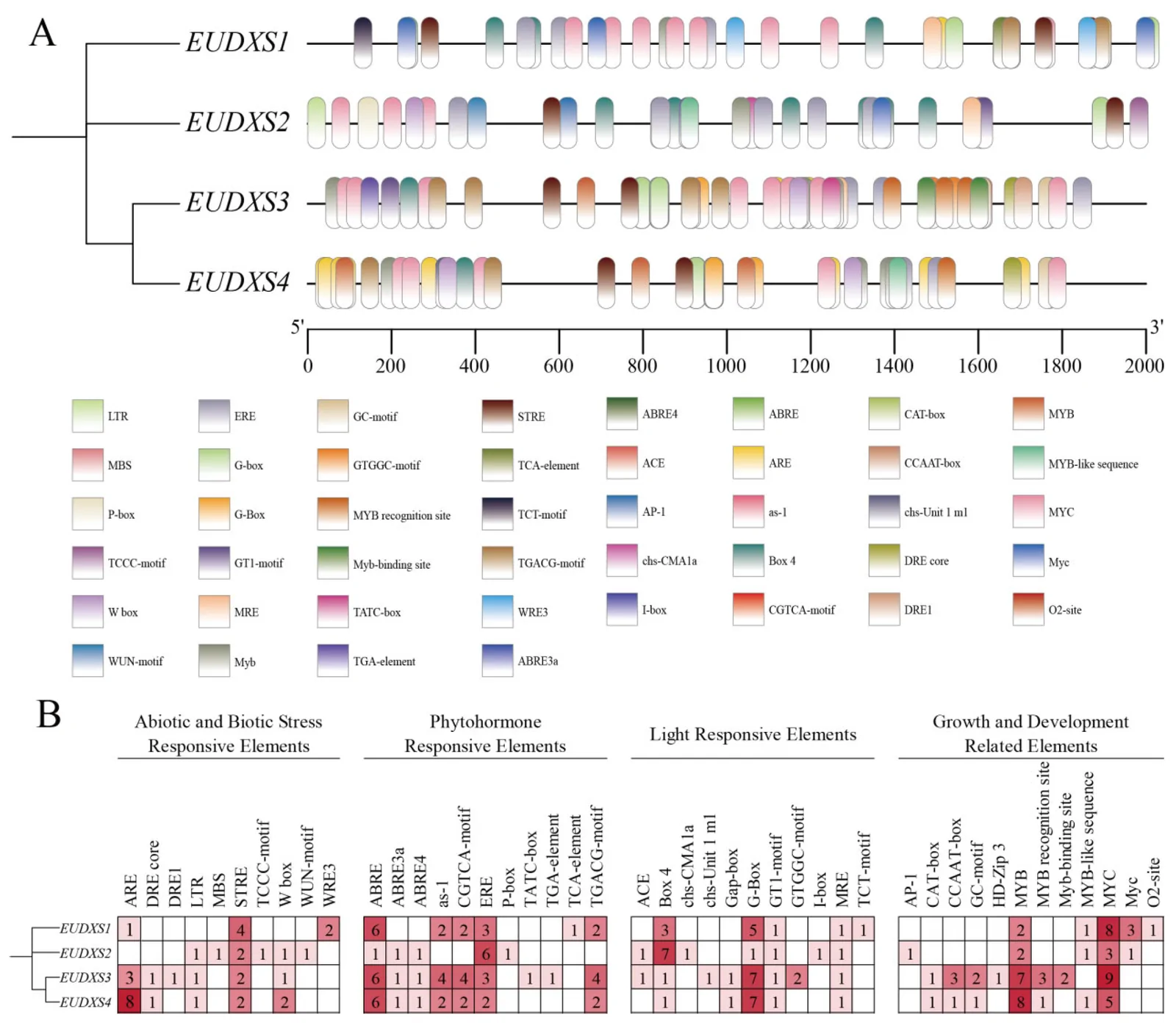
*Cis-acting* elements in *EuDXS* promoters. (**A**) Map of the location of *cis-acting* elements. (**B**) Heatmap illustrating the number of promoter elements.

*Cis-acting* elements of promoter serve as binding sites for transcription factors and regulate gene expression. PlantCARE was used to predict the *cis-acting* elements of *EuDXS* genes by extracting the 2000-bp upstream promoter regions of *EuDXS* genes (Figure 4A). The *cis-acting* elements were classified into four categories: 37 elements related to abiotic and biotic stress, 67 elements related to phytohormone regulation, 50 elements associated with light responsiveness and 69 elements related to growth and development processes (Figure 4B).

In abiotic and biotic stress elements, ARE had the largest quantity of 12, followed by STRE (10), suggesting that *EuDXS* genes may participate in anaerobic regulation. For phytohormone regulation elements, 25 abscisic acid (ABA) responsive elements including ABRE, ABRE3a and ABRE4 were detected, among which ABRE was the most numerous and existed in four genes. A total of 16 methyl jasmonate responsive elements (TGACG-motif, CGTCA-motif) were identified; fourteen ethylene-responsive elements existed in all members. It could be inferred that *EuDXS* genes were involved in multiple phytohormones response. G-box was the most abundant light responsive element (20), followed by Box4 (12), indicating that *EuDXS* gene expression may be modulated by photoperiod. MYC element ranked first (29) among growth and development process.

### 2.5. Expression Pattern of EuDXS Genes

**Figure 5:**
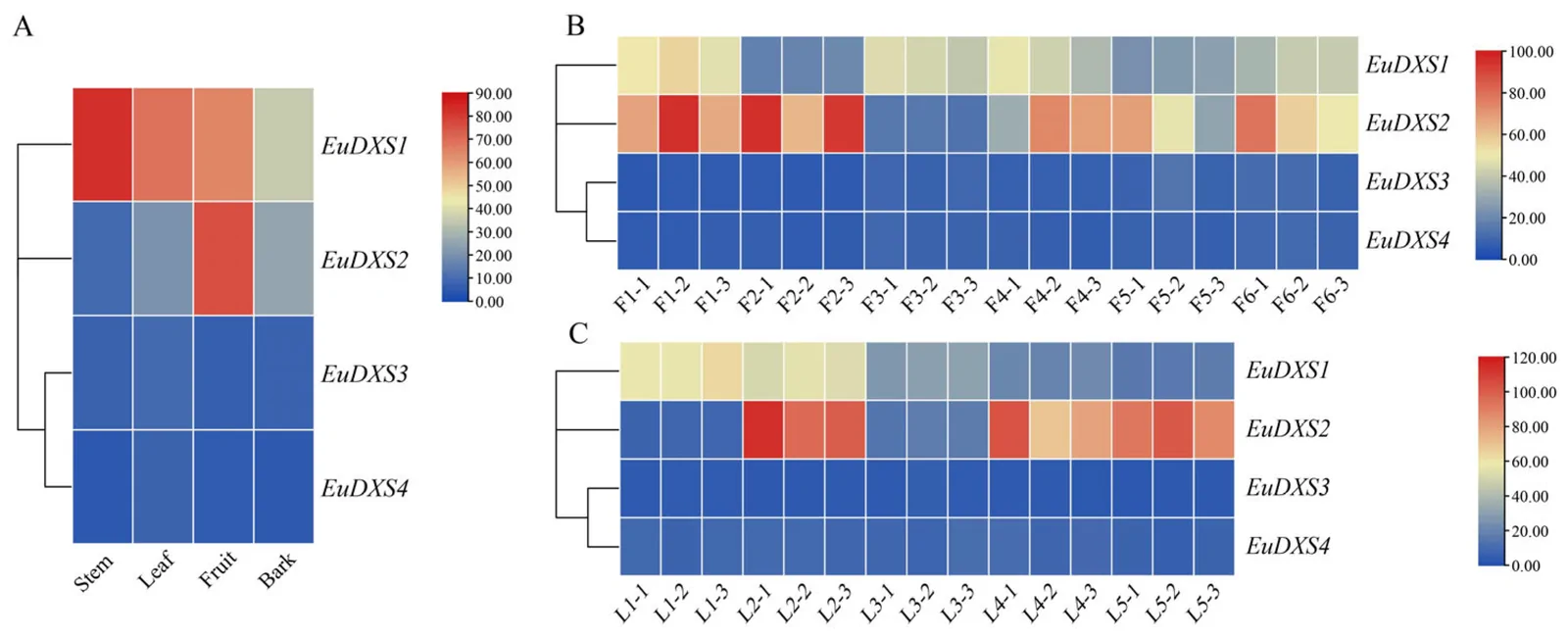
Expression pattern of *EuDXS* genes in different tissues (**A**) and developmental stages of fruits (**B**) and leaves (**C**). Note: F1 to F6 represents six developmental stages of fruits; L1 to L5 represents five developmental stages of leaves. -1/-2/-3 represents three biological replicates. Color scale indicates gene expression levels, red represents high expression and blue represents low expression.

**Figure 6:**
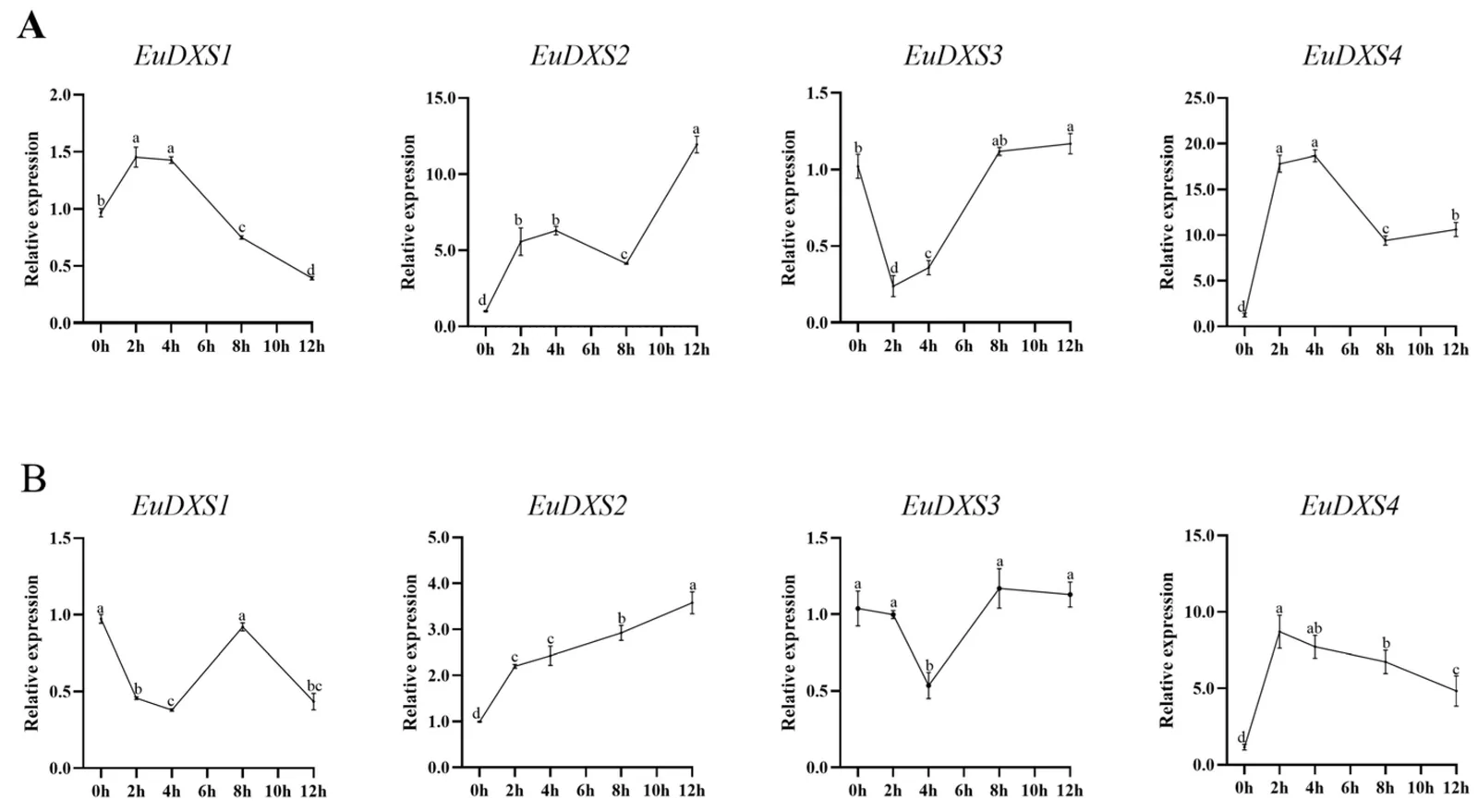
Expression pattern of *EuDXS* genes under salt (**A**) and drought (**B**) stress conditions. Note: The horizontal axis represents the treatment time. Different letters represent the significant of differences (*p* < 0.05).

To elucidate the function of *EuDXS* genes, expression profiles of *EuDXSs* were further analyzed in different tissues, under salt and drought stress treatment and at different developmental stages of fruits and leaves. The results showed that the expression of *EuDXS* genes were varied among the stem, leaf, fruit and bark of *E. ulmoides* (Figure 5A). *EuDXS1* exhibited the highest expression in stem, followed by fruit and leaf. *EuDXS2* was primarily expressed in fruit, with expression levels significantly higher than that in other tissues. In contrast, *EuDXS3* and *EuDXS4* exhibited low expression levels in different tissues. As shown in Figure 5B, there were no significant differences in the expression levels of *EuDXS3* and *EuDXS4* across the six developmental stages of *E. ulmoides* fruits, and their expression levels were significantly lower than those of *EuDXS1* and *EuDXS2*. Except for the third stage, the expression level of *EuDXS2* was higher than that of other three genes across different developmental stages. Similar to the expression patterns in fruits, *EuDXS3* and *EuDXS4* also exhibited low expression levels at different developmental stages of leaves. *EuDXS1* was highly expressed at the first two developmental stages, while *EuDXS2* reached its peak expression at the second stage (Figure 5C). Moreover, both *EuDXS1* and *EuDXS2* exhibited high expression levels during the early developmental stages of fruits and leaves, which is a critical period for *E. ulmoides* rubber synthesis. It is speculated that *EuDXS1* and *EuDXS2* may play important roles in the biosynthesis of *E. ulmoides* rubber. The qRT-PCR analysis revealed that *EuDXS* genes responded differently to salt and drought stress (Figure 6). The expression levels of *EuDXS1*, *EuDXS2* and *EuDXS4* exhibited a trend of first increasing and then decreasing, *EuDXS1* and *EuDXS4* expression levels rose rapidly 2 h after the salt stress treatment (Figure 6A). Under drought stress, the expression levels of *EuDXS1* and *EuDXS3* both exhibited a trend of ‘decrease-rise-decrease’. *EuDXS2* remained consistently upregulated, while *EuDXS4* showed an expression pattern of initial upregulation followed by downregulation (Figure 6B).

### 2.6. Transient Expression in Tobacco

**Figure 7:**
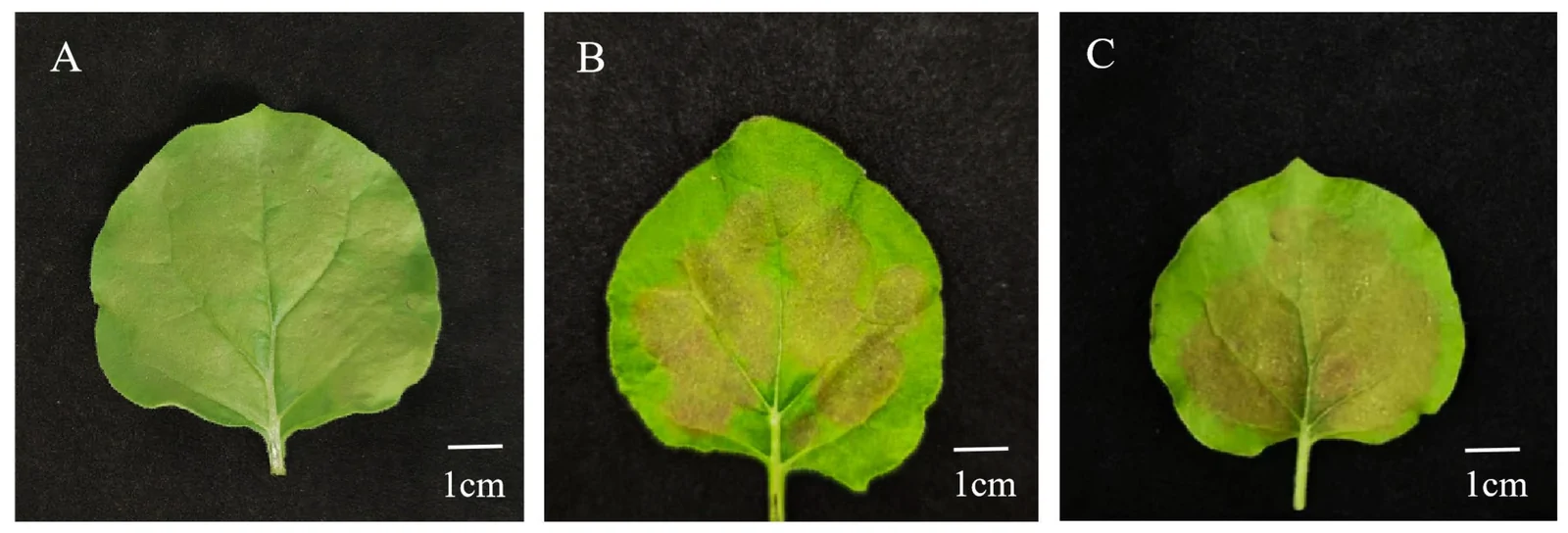
Phenotypes following transient expression of *EuDXS1* and *EuDXS2* genes in tobacco leaves. (**A**) CK. (**B**) OE1. (**C**) OE2. Note: CK represents empty pCAMBIA1301-RUBY vector. OE1 and OE2 represent overexpression of *EuDXS1* and *EuDXS2*, respectively. The same as below.

**Figure 8:**
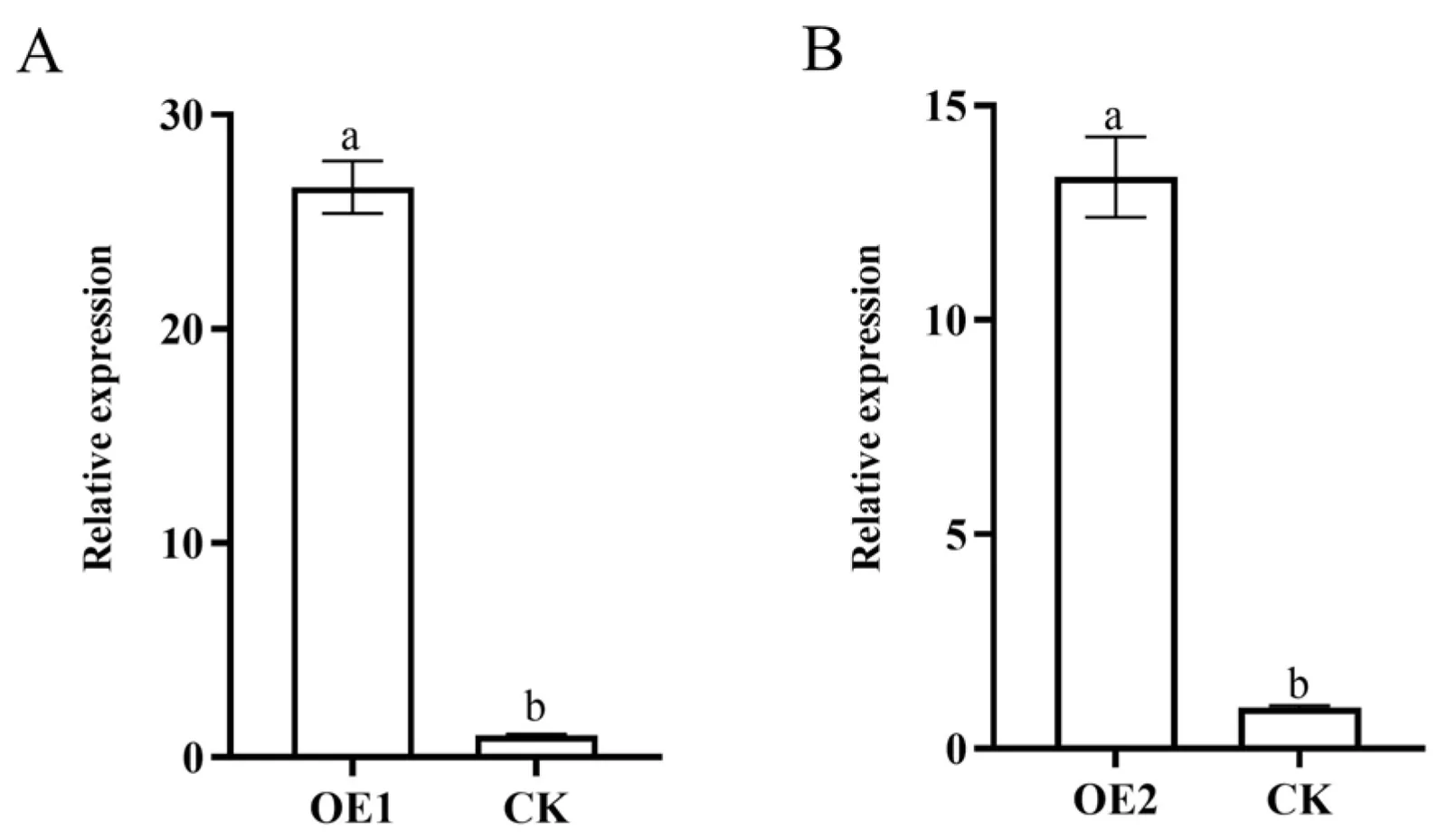
Relative expression levels of *EuDXS1* (**A**) and *EuDXS2* (**B**) between CK and transiently transformed tobacco. Different letters represent the significant of differences (*p* < 0.05).

**Figure 9:**
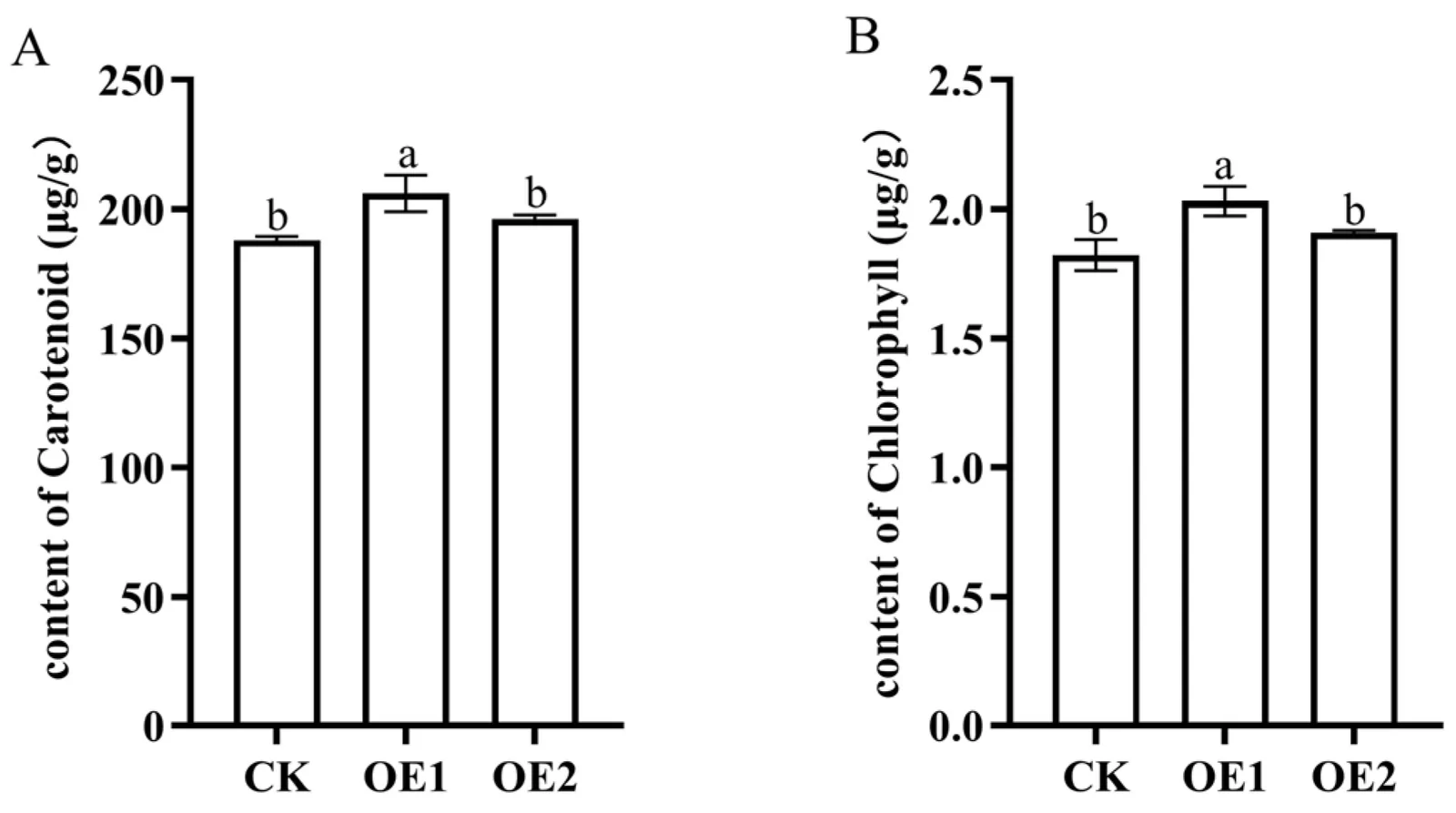
Determination of carotenoid (**A**) and chlorophyll (**B**) content in CK and transiently transformed tobacco leaves. Different letters represent the significant of differences (*p* < 0.05).

Considering that *EuDXS1* and *EuDXS2* exhibited substantially higher transcript levels compared with *EuDXS3* and *EuDXS4*, and both showed high expression in tissues and early-developmental stages closely associated with *E. ulmoides* rubber biosynthesis. Therefore, *EuDXS1* and *EuDXS2* were prioritized for further transient expression in tobacco. The results showed that overexpressed *EuDXS1* and *EuDXS2* had a deeper color than the control group (Figure 7). The expression analysis results showed that both *EuDXS1* and *EuDXS2* were significantly expressed in transiently transformed tobacco leaves (Figure 8). We further determined the content of carotenoid and total chlorophyll, in *EuDXS1* transiently transformed leaves, the carotenoid and total chlorophyll contents were significantly increased compared with CK, whereas no significant difference in these two compounds was observed between *EuDXS2* and CK (Figure 9).

## 3. Discussion

*E. ulmoides* rubber is a typical polyterpene compound formed by condensation of isopentenyl pyrophosphate (IPP) and dimethylallyl pyrophosphate (DMAPP). The synthesis of IPP relies on the MEP and MVA pathways. As the initial key enzyme in the MEP pathway, *DXS* is a vital regulatory gene that modulates the supply of terpenoid precursors and promotes the efficient biosynthesis of *E. ulmoides* rubber [18,19]. Studies have shown that most plants contain more than one *DXS* gene. Specifically, 7 *DXS* genes have been identified in *Lycium barbarum* [10], 22 *DXS* genes in *Lavandula angustifolia* [20], and 5 *DXS* genes in *Glycyrrhiza uralensis* [21], indicating that the number of *DXS* gene family members varies significantly among different species. In this study, a total of four *EuDXS* genes were identified through genome-wide analysis, all of which were found to contain conserved domains and functional modules, laying a foundation for the functional diversification of the *EuDXS* family in *E. ulmoides*. The proteins encoded by *EuDXS* genes were hydrophilic and localized to the chloroplasts. Similarly, four AaDXS proteins of *Artemisia annua* and three PgDXS proteins of *Platycodon grandiflorum* are also localized to the chloroplast [22,23], which is consistent with the plastidial localization of the MEP pathway, confirming that the DXS proteins are synthesized in the cytoplasm and participate in the biosynthesis of terpenoids. Phylogenetic analysis among *E. ulmoides*, *Arabidopsis thaliana*, *Solanum lycopersicum* and ‘84K’ poplar revealed that *EuDXS* family members exhibited distinct branches and were classified into three subfamilies, which is consistent with those determined in most plants, such as *Glycyrrhiza uralensis* [21] and tea plant [24]. EuDXS1 and EuDXS2 belonged to Group I and Group II, respectively, while EuDXS3 and EuDXS4 clustered into Group III, indicating that *EuDXS* genes possessed diverse functions. Genes located on adjacent chromosomes generally exhibit high homology. Both *EuDXS3* and *EuDXS4* genes were located on chromosome 5, suggesting that they shared a high degree of homology and similar biological functions, which is in accordance with the findings of *DXS* gene family members in *Lycium barbarum* [10] and *Glycyrrhiza uralensis* [21].

The promoter is a specific DNA sequence located approximately 2000 bp upstream of the start codon, containing various *cis-acting* elements that assist plants in responding to exogenous hormones and environmental stress [25,26]. In this study, the promoters of most *EuDXS* genes exhibited a variety of *cis-acting* elements, such as stress-responsive elements, hormone-responsive elements, light-responsive elements, growth and development-responsive elements. Among the hormone-responsive elements, ABA- and ethylene-related elements were detected in all four members. Relevant studies have shown that ABA-related elements could influence terpenoid synthesis by regulating *DXS* gene expression [27]. In rubber trees, ethylene regulates latex-related gene expression in a dose-dependent manner by modulating *ERF*, thereby increasing rubber yield [28]. We hypothesize that *DXS* genes may be involved in responding to exogenous hormones, thereby promoting the biosynthesis of rubber-related substances. As a crucial environmental factor, light not only provides energy for photosynthesis, but also acts as a key signal regulating the entire plant life cycle and secondary metabolic pathways [29]. G-Box was the most abundant in the light-responsive element, indicating that *EuDXS* genes are involved in photoperiod response. Previous studies have shown that shading treatment significantly suppressed *DXS* expression and disrupted downstream terpenoid synthesis in fruits of peach and tomato [30,31]. Furthermore, qRT-PCR analysis indicated that *EuDXS* genes responded significantly to salt and drought stress. Studies revealed that transgenic tobacco with *DXS* overexpression exhibited markedly enhanced tolerance to drought and salt stress [12]. These results indicate that the expression of *EuDXS* genes in *E. ulmoides* is induced by multiple factors, thereby regulating terpenoid biosynthesis and responding to environmental stress.

Like most gene families, *EuDXS* genes exhibited differential expression patterns across various tissues and developmental stages in *E. ulmoides*. Previous studies have indicated that *Eucommia* rubber is mainly synthesized in pericarp, with its content remarkably higher than that in other tissues [32]. To investigate the *EuDXS* genes involved in the biosynthesis of *E. ulmoides* rubber, this study analyzed the expression pattern of *EuDXS* genes in fruits and leaves at different developmental stages, as well as in various tissues based on transcriptome data. Among different tissues, *EuDXS3* and *EuDXS4* showed relatively low expression levels, while *EuDXS1* was predominantly expressed in leaf, stem and fruit, *EuDXS2* was mainly expressed in fruit. Further analysis of *EuDXS* gene expression levels at different developmental stages of fruits and leaves demonstrated that *EuDXS1* and *EuDXS2* were highly expressed at the early stage compared to other periods and family members. Previous studies have shown that the early developmental stages of fruits and leaves are critical for the rapid synthesis of *E. ulmoides* rubber [33]. However, *EuDXS3* and *EuDXS4* exhibited relatively low transcript abundance across most tested tissues and developmental stages. Low constitutive expression of these two genes may indicate that they are not the main contributors to the basal terpenoid supply under normal growth conditions, they may function under very specific conditions, such as particular stress treatments. The qRT-PCR analysis revealed that within the first eight hours of salt and drought stress treatments in *E. ulmoides* seedlings, *EuDXS3* exhibited a trend of first decreasing and then increasing, whereas *EuDXS4* showed a pattern of first increasing and then decreasing under both stress conditions. In the present study, a transient expression system in *Nicotiana benthamiana* was adopted for functional characterization of *EuDXS1* and *EuDXS2*. qRT-PCR verified that the transcript levels of target genes were markedly higher in *EuDXS1* and *EuDXS2* transient expression leaves than that in CK, confirming the reliability of this transient transformation system for preliminary gene-function assay. In addition, overexpression of *EuDXS1* significantly increased total chlorophyll and carotenoid contents, while overexpression of *EuDXS2* showed no obvious effect on photosynthetic pigments. As the first rate-limiting enzyme of the MEP pathway, overexpression of *DXS* enhances the accumulation of plastid-derived terpenoids such as chlorophyll, carotenoids, partial monoterpenes and diterpenoids in many plant species. Heterologous overexpression of *PmDXS* in tobacco significantly elevated chlorophyll and carotenoid contents, and overexpression of *SmDXS2* promoted diterpene tanshinone production in *Salvia miltiorrhiza* hairy roots [34,35]. In summary, it can be concluded that *EuDXS1* and *EuDXS2* may be involved in terpenoid biosynthesis of *E. ulmoides*.

## 4. Materials and Methods

### 4.1. Plant Materials and Growth Conditions

The fruits and leaves of *E. ulmoides* were obtained from the Yuanyang Test Base, Research Institute of Non-timber Forestry, Chinese Academy of Forestry. Fruit samples were collected starting on April 20, with sampling performed every 30 days for a total of six time-points, which were designated F1–F6. Leaf samples were collected starting on April 10, with sampling performed every 30 days for a total of five time-points, which were designated L1–L5. All samples were subjected to three biological replicates and were immediately frozen in liquid nitrogen and stored in an ultra-low temperature refrigerator at −80 °C for transcriptome sequencing.

Two-month-old *E. ulmoides* seedlings with consistent growth were subjected to salt stress (200 mmol·L^−1^ NaCl, Tianjin, China) and drought stress (20% PEG-6000, Tianjin, China) treatments. Leaves were collected at five time points including 0 h, 2 h, 4 h, 8 h and 12 h after treatment, then immediately frozen in liquid nitrogen for total RNA extraction.

Seeds of *Nicotiana benthamiana* L. were sown in peat in seedling trays and placed in a growth chamber maintained at 25 °C and 50% relative humidity under a 16-h light/8-h dark cycle. Once the seedlings developed three to four true leaves, they were transferred to larger trays and cultured for 5 to 6 weeks for Agrobacterium-mediated transient expression.

### 4.2. Genome-Wide Identification of EuDXS Gene Family

Genome data and DXS protein sequences of *E. ulmoides* were provided by our research group [36]. BLASTp (sequence identity threshold > 70%) and Simple HMM search (E < 1 × 10^−5^) function in TBtools‑II (v2.390) were used to screen *DXS* gene family members from the *E. ulmoides* genome. The sequences of candidate members were uploaded to InterPro (https://www.ebi.ac.uk/interpro/, accessed on 15 May 2026) to verify the presence of the conserved domain DXP, and only gene family members with the *DXP* conserved domain were retained. The pfam model was downloaded from the InterPro website to identify the PF number of the *DXS* gene, and PF13292 was selected for HMMER analysis. The protein sequences of candidate members were uploaded to the SMART website (https://smart.embl.de/, accessed on 16 May 2026) to identify conserved protein sequences of the candidates, and only members with characteristic DXS proteins were retained. The *DXS* gene family members were ultimately determined by integrating results from BLASTp and HMMER.

### 4.3. Physicochemical Property and Phylogenetic Relationship Analysis

The Protein Parameter Calc function of TBtools was used to analyze the amino acid number, molecular weight, isoelectric point, instability index and aliphatic index of *EuDXS* gene members. In silico subcellular localization prediction was performed using the Cell-PLoc website (http://www.csbio.sjtu.edu.cn/bioinf/Cell-PLoc/, accessed on 20 May 2026). Protein secondary structure prediction was carried out using the NPS@ website (https://npsa.lyon.inserm.fr/cgi-bin/npsa_automat.pl?page=/NPSA/npsa_sopma.html, accessed on 20 May 2026).

The phylogenetic evolutionary tree of the *DXS* gene members from *E. ulmoides*, *Arabidopsis thaliana*, *Solanum lycopersicum* and ‘84K’ poplar was constructed using the MEGA-X software (v10.2.1). Genome data and DXS protein sequences of *E. ulmoides* were provided by our research group. The genome data and DXS protein sequences of *Arabidopsis thaliana*, ‘84K’ poplar and *Solanum lycopersicum* were downloaded from TAIR (https://www.arabidopsis.org/, accessed on 15 May 2026), NCBI (https://www.ncbi.nlm.nih.gov/, accessed on 15 May 2026) and Ensembl (https://www.ensembl.org/index.html?redirect=no, accessed on 15 May 2026), respectively. The Gene Location Visualize from GTF/GFF function embedded in TBtools was adopted to map the chromosomal distribution of *EuDXS* family members.

### 4.4. Gene Structure and Promoter Cis-Acting Elements Analysis

The MEME-Suite(v5.5.1, http://meme-suite.org/tools/meme, accessed on 26 May 2026) was applied to identify conserved motifs. The domain analysis was carried out using the NCBI-CDD tool (https://www.ncbi.nlm.nih.gov/Structure/cdd/wrpsb.cgi, accessed on 26 May 2026). All results were visualized leveraging TBTools.

The 2000-bp upstream promoter regions of *EuDXS* genes were extracted using TBtools, and then *cis-acting* elements analysis was performed with PlantCARE (https://bioinformatics.psb.ugent.be/webtools/plantcare/html/, accessed on 29 May 2026).

### 4.5. Expression Pattern of EuDXS Genes

Transcriptome sequencing of fruits and leaves was performed by Tianjin Jizhi Gene Technology Co., Ltd. (Tianjin, China). The transcriptome data of different tissues (stem, leaf, fruit and bark) was downloaded from NCBI (PRJNA792509). The relative expression profiles of *EuDXS* genes were evaluated, and the heatmap was constructed by TBtools software. Real-time fluorescence quantitative PCR (qRT-PCR) reaction was performed to detect expression level of *EuDXS* genes under salt and drought stress. The *Actin* was used as the reference gene. The data was analyzed via the 2^-△△Ct^ method, and graphs were plotted using SigmaPlot 12.5 software. The primer sequences are shown in Table 3. Relative gene-expression data were subjected to one-way analysis of variance (ANOVA), followed by Duncan multiple-comparison test for pairwise comparisons among groups.

### 4.6. Transient Expression in Tobacco

Based on the results of expression pattern analysis, *EuDXS1* and *EuDXS2* were selected as candidate genes. The target full-length native coding-sequence fragments of *EuDXS1* and *EuDXS2* were generated by gene synthesis based on their reference gene sequences. No codon optimization was performed, and the predicted N-terminal chloroplast transit peptide was fully retained in the synthetic fragments. These synthetic fragments were assembled into pCAMBIA1301-RUBY vector. The primers used for vector construction are shown in Table S1. The recombinant plasmid was transformed into *Agrobacterium tumefaciens* GV3101 cells. The bacterial suspension was grown at 28 °C in selective YEB medium supplemented with rifampicin and kanamycin. After shaking for 36h, agrobacterium cells were harvested through centrifugation, resuspended in infiltration buffer to a final OD600 of 1.0. After 3 h of incubation, the agrobacterium was infiltrated into the leaves with a micro syringe. Tobacco leaves infiltrated with *A. tumefaciens* harboring empty pCAMBIA1301-RUBY vector were used as the control (CK). The phenotypic observation and leaf RNA extraction were performed 2 days after infiltration. Then the expression analysis of *EuDXS1* and *EuDXS2* was conducted by qRT-PCR. The primer sequences are shown in Table S2. The *GAPDH* was used as the reference gene. Further we determined the content of carotenoid and chlorophyll content in CK and transiently transformed tobacco leaves, and the methods were followed aas described by Li et al. [34].

## 5. Conclusions

The key role of the *DXS* genes in plant growth and development and terpenoid biosynthesis has been well elucidated in many plant species, however, no studies on *DXS* gene family in *E. ulmoides* have been reported yet. Here, we conducted a genome-wide identification and expression of *DXS* family members in *E. ulmoides*. A total of four *EuDXS* genes were identified. Phylogenetic and gene structure analysis revealed that *EuDXS* family members can be divided into three subfamilies, which were unevenly distributed on three chromosomes. Expression pattern analysis indicated that *EuDXS1* and *EuDXS2* might play specific roles in the growth and rubber synthesis of *E. ulmoides*. Transient expression in tobacco confirmed that *EuDXS1* and *EuDXS2* could increase the content of terpenoid substances such as total chlorophyll and carotenoid. Our findings will provide theoretical reference for further exploring the molecular characteristics and biological functions of *EuDXS* genes.

## Figures and Tables

**Table 1:** Detailed physicochemical property of EuDXS proteins.

Gene Name	Gene ID	Number of Amino Acid	Molecular Weight/kDa	Theoretical pI	Instability Index	Aliphatic Index	GRAVY	In Silico Subcellular Localization
*EuDXS1*	*Eu0120438.1*	692	75.37	8.69	40.80	85.29	−0.196	chlo
*EuDXS2*	*Eu0127987.1*	713	76.61	6.79	37.34	91.29	−0.052	chlo
*EuDXS3*	*Eu0108829.1*	625	68.01	7.96	35.43	86.16	−0.067	chlo
*EuDXS4*	*Eu0108841.1*	625	67.98	7.03	35.53	86.78	−0.057	chlo

**Table 2:** Secondary structure analysis of *EuDXS* gene family.

Gene Name	Alpha Helix		Extended Strand		Random Coil	
	Quantity	Proportion	Quantity	Proportion	Quantity	Proportion
*EuDXS1*	277	40.03%	90	13.01%	325	46.97%
*EuDXS2*	270	37.87%	90	12.62%	353	49.51%
*EuDXS3*	232	37.12%	89	14.24%	304	48.64%
*EuDXS4*	231	36.96%	81	12.96%	313	50.08%

**Table 3:** Primer sequences of *EuDXS* genes.

Gene Name	Forward (5′-3′)	Reverse (5′-3′)
*EuDXS1*	TGGCTGTGGGAAGGGATCTA	CCGGCATTGTTCATGGCTTC
*EuDXS2*	AAGGTTTCCGGATCGCTGTT	TGAGTAGATGGCGCAGAACG
*EuDXS3*	TTTCTCCCAAAGTGCAGTTT	GCGGTCCTTGAAATTAAGTG
*EuDXS4*	TTTCTCCCAAAGTGCAGTTC	GCGTTCCTTGAAATTAAGTA
*Actin*	TTGTTAGCAACTGGGATGATATGG	CAGGGTGTTCTTCAGGAGCAA

## Data Availability

The data presented in this study are included in the article/Supplementary Materials. Further inquiries can be directed to the corresponding author.

## Supplementary Materials

The following supporting information can be downloaded at: https://www.mdpi.com/article/10.3390/plants15182843/s1, File S1: The CDS sequences of four *EuDXS* genes. File S2: The genomic sequences of four *EuDXS* genes. Table S1: Primer sequences of vector construction. Table S2: Primer sequences used in transient expression.

## References

[1] Li C., Zha W., Li W., Wang J., You A. (2023). Advances in the Biosynthesis of Terpenoids and Their Ecological Functions in Plant Resistance. Int. J. Mol. Sci..

[2] Du H., Hu W., Yu R. (2013). China Eucommia Rubber Resources and Industry Development Report (2013).

[3] Jin C., Li Z., Li Y., Wang S., Li L., Liu M., Ye J. (2020). Transcriptome Analysis of Terpenoid Biosynthetic Genes and Simple Sequence Repeat Marker Screening in *Eucommia ulmoides*. Mol. Biol. Rep..

[4] Bamba T., Murayoshi M., Gyoksen K., Nakazawa Y., Okumoto H., Katto H., Kobayashi A. (2010). Contribution of mevalonate and methylerythritol phosphate pathways to polyisoprenoid biosynthesis in the rubber-producing plant *Eucommia ulmoides* Oliver. Z. Für Naturforschung C.

[5] Lange B.M., Rujan T., Martin W., Croteau R. (2000). Isoprenoid biosynthesis: The evolution of two ancient and distinct pathways across genomes. Proc. Natl. Acad. Sci. USA.

[6] Sprenger G.A., Schörken U., Wiegert T., Grolle S., Graaf A.A.D., Taylor S.V., Begley T.P., Bringer-Meye S., Sahm H. (1997). Identification of a thiamin-dependent synthase in *Escherichia coli* required for the formation of the 1-deoxy-d-xylulose-5-Phosphate precursor to isoprenoids, thiamin, and pyridoxol. Proc. Natl. Acad. Sci. USA.

[7] Estévez J.M., Cantero A., Reindl A., Reichler S., Leon P. (2001). 1-deoxy-d-xylulose-5-Phosphate synthase, a limiting enzyme for plastidic isoprenoid biosynthesis in plants. J. Biol. Chem..

[8] Zhang L., Zhang Z., Zheng T., Wei W., Zhu Y., Gao Y., Yang X., Lin S. (2016). Characterization of carotenoid accumulation and carotenogenic gene expression during fruit development in yellow and white loquat fruit. Hortic. Plant J..

[9] Di X., Rodriguez-Concepcion M. (2023). Exploring the deoxy-D-xylulose-5-phosphate synthase gene family in tomato (*Solanum lycopersicum*). Plants.

[10] Qi C., Zhang L., Wang B., Cao J., Ma Y., Cao B. (2025). Genome-wide identification and expression analysis of *LbDXS* gene family in *Lycium barbarum*. J. Cent. S. Univ. For. Technol..

[11] Zhang M. (2008). Identification and Characterization of Genes Encoding Key Enzymes in Soybean Non-Mevalonate Pathway. Master’s Thesis.

[12] Zhao H., Su J., Zhong Z., Xiong T., Dai W., Zhang D., Chang Y. (2024). Functional identification and regulatory active site screening of the DfDXS gene of Dryopteris fragrans. Plants.

[13] Mo J., Zhao D. (2014). The relationship between the expression of isoprene metabolic pathway genes and gutta-percha rubber contents in *Eucommia ulmoides* Oliver. J. Mt. Agric. Biol..

[14] Wang L., Du H., Wuyun T. (2016). Genome-wide identification of microRNAs and their targets in the leaves and fruits of *Eucommia ulmoides* using high-throughput sequencing. Front. Plant Sci..

[15] Chen J., Zhao D.G. (2004). Research advances on the enzymes and their coding gene involved in plant terpene biosynthesis. Mol. Plant Breed..

[16] Kim B.R., Kim S.U., Chang Y.J. (2005). Differential expression of three 1-deoxy-D-xylulose-5-phosphate synthase genes in rice. Biotechnol. Lett..

[17] Zeng S., Gao T., Gu M., Hou B., Ren W., Zhang Y., Ye N. (2024). Genome-wide identification and expression of the DXS gene family in Huangdan (*Camellia sinensis*). Chin. J. Appl. Environ. Biol..

[18] Chen D., Ye H., Li G., Liu Y. (2000). Advances in molecular biology of plant isoprenoid metabolic pathways. Acta Bot. Sin..

[19] Ganjewala D., Kumar S., Luthra R. (2009). An account of cloned genes of methyl-erythritol-4-phosphate pathway of isoprenoid biosynthesis in plants. Curr. Issues Mol. Biol..

[20] Kelaremu K., Chen Y., Li C., He Q., Chen L. (2023). Genome-wide identification and expression analysis of the DXS gene family in *Lavandula angustifolia*. Genom. Appl. Biol..

[21] Sun Y., Ouyang X., Ma H., Shi N., Yao H., Shen H. (2025). Identification of the *DXS* gene family in licorice and its expression analysis under abiotic stress. Plant Physiol. J..

[22] Liu W.H. (2016). Study on Functional Differentiation of *AaDXS* Gene Family and Molecular Mechanism of Low Temperature Improving Artemisinin Production in *Artemisia annua* L.. Ph.D. Thesis.

[23] Dong N., Yu H., Liu M.L., Li J., Chen B.W., Chang X.W., Wang J.T., Cha L.P., Gui S.Y. (2023). Prokaryotic expression, subcellular localization and enzyme activity analysis of *DXS* gene from *Platycodon grandiflorum*. Acta Pharmacol. Sin..

[24] Zhang W.H. (2018). Expression, Cloning of *DXS* and *DXR* Genes in Tea Plant and Their Response to Exogenous Potassium. Master’s Thesis.

[25] Liu J., Chen Y., Liu Y., Si F. (2021). Identification and expression analysis of *TIFY* transcription factors in *Eucommia ulmoides*. Chin. J. Exp. Tradit. Med. Formulae.

[26] Chen J., Lu C., Wang G., Li C., Li Y., Su F., Wang C., Zhang Y. (2023). Cloning and expression analysis of U6 promoter in *Panax Quinquefolius*. China J. Chin. Mater. Medica.

[27] Dabiri M., Majdi M., Bahramnejad B. (2020). Partial sequence isolation of *DXS* and *AOS* genes and gene expression analysis of terpenoids and pyrethrin biosynthetic pathway of *Chrysanthemum cinerariaefolium* under abiotic elicitation. Acta Physiol. Plant..

[28] Wan J., Fan R., Yang W., Wei F., Gao H., Wei H., Qiu J. (2024). New insights into ethylene-induced latex flow in a dose-dependent manner in rubber tree. Ind. Crops Prod..

[29] Zhu M.Y. (2016). Effect of Light on Alkaloid Synthesis and Gene Expression Levels in *Catharanthus roseus* Seedlings. Master’s Thesis.

[30] Dong X.T. (2015). Effect of Shading Bagging on Quality Traits Such as Carotenoid Accumulation and Related Gene Expression in Yellow Peach Fruit. Master’s Thesis.

[31] Li X.M. (2020). Study on the Role of HY5 in Carotenoid Synthesis in Tomato Fruit. Master’s Thesis.

[32] Xie B., Du H., Du L., Fu J. (2005). Variations of gutta-percha content in samara from different *Eucommia ulmoides* forms. Sci. Silvae Sin..

[33] Wuyun T., Wang L., Liu H., Wang X., Zhang L., Bennetzen J.L., Li T., Yang L., Liu P., Du L. (2018). The hardy rubber tree genome provides insights into the evolution of polyisoprene biosynthesis. Mol. Plant.

[34] Liu Y., Wang Y., Wang X., Li Y. (2021). Characterization and function of the 1-deoxy-D-xylose-5-phosphate synthase (DXS) gene related to terpenoid synthesis in *Pinus massoniana*. Int. J. Mol. Sci..

[35] Wu Y., Zhou J., Li M., Wang G. (2010). Molecular cloning and characterization of two 1-deoxy-D-xylulose-5-phosphate synthase genes involved in tanshinone biosynthesis in *Salvia miltiorrhiza*. J. Exp. Bot..

[36] Du Q., Wu Z., Liu P., Qing J., He F., Du L., Sun Z., Zhu L., Zheng H., Sun Z. (2023). The chromosome-level genome of *Eucommia ulmoides* provides insights into sex differentiation and α-linolenic acid biosynthesis. Front. Plant Sci..

