# Supplementary material for: Genome-Wide Identification and Expression Pattern Analysis of the DXS Gene Family in Eucommia ulmoides
Source: Plants (Basel). 2026 Sep 17;15(18):2843. doi: 10.3390/plants15182843 (PMC13610960; doi:10.3390/plants15182843)
Supplement: Supplementary file 1 [file plants-15-02843-s001.zip › File S1 The CDS sequences of four EuDXS genes.pdf]

>EuDXS1
ATGGCGCTCTGTACATATTCTTCATTCCCTGGACATTTTAACAGAGCTGTGGTGGCTTCA
GATCTCCAAAAGCAATTTGGTTTTTGTTCCAAGGGGAAAGATGTTCACCAAAAACATACC
CAGCTCAAGAAAAGATCAATTGGGGTCTGGGCATCTCTATCGGAAACAGGGGAATATCAC
TCCAAAAGACCTCCAACCCCACTTTTGGACACTATCAACTATCCAATTCATATGAAGAAT
CTCTCCATCAAGGAACTGAAACAACTCGCTGAGGAGGTGCGATCTGATGTGATCTTCAAT
GTTTCCAAGACCTGGCGGTCACCTCGAATTCTCTGGGATGTTGGTCATCAGTCTTACCCA
CACAAGATTTTGACTGGGAGAAGAGACAAAATGCATACGATTAGACAGACGAACGGGCTT
GCTGGATTCACTAAGCGCTCTGAGAGCGAGTATGATTGCTTCGGCACAGGTCACAGTTCT
ACCACAATCTCAGCAGGCTTAGGGATGGCTGTGGGAAGGGATCTAAAGCGAAGAAAGAAC
AATGTAGTTTCCGTGATAGGCGACGGTGCAATGACGGCAGGTCAGGCTTATGAAGCCATG
AACAATGCCGGCTACCTCGATTCAGACATGATTGTCATCCTTAACGACAACAAACAGGTT
TCATTACCAACCGCCAACCTCGATGGGCCCATTCCACCCGTCGGAGCTTTGAGCTCTGCT
CTCAGTAGATTGCAGTCCAACCGGCCTCTCCGTGAACTTAGAGAAGTAGCCAAGGGAGTT
ACGAAGCAAATCGGGTGGCCTATGCACGATCTAGCATCGAAAGTCGACGAATATGCTCGC
GGCCTAATAAGCGGTTCTGGATCGACTCTATTTGAAGAGCTTGGACTTTATTATATCGGT
CCTGTAGACGGTCACAGCATAGATGATCTTGTTGCAATTCTTAAGGAAGTCAAGAGTACT
CGAACAACCGGCCCTGTTTTAATCCATGTTGTCACCGAGAAAGGAAGAGGATACCCTTAC
GCTGAGAAAGCCGCCGACAAGTACCACGGAGTGGCCAAGTTTGATCCGGCTACAGGACAG
CAATTCAAAGCTAGTGTCAAGACTCAATCTTTCACAACATATTTTGCCGAGGCTTTGATT
GCTGAGGCAGAAGCCGATAAAGATGTAGTTGCGATTCACGCGGCAATGGGAGGAGGAACC
GGTTTGAATCTCTTCCTACGTAGATTCCCAACTCGGTGTTTCGACGTGGGGATAGCCGAA
CAACACGCGGTCACTTTTGCCGCCGGTTTGGCCTGTGAAGGACTAAAACCCTTCTGCGCT
ATTTATTCTTCTTTCATGCAGAGAGCTTATGATCAGGTCGTGCACGACGTCGACTTGCAA
AAGCTTCCGGTAAGATTTGCAATGGACCGGGCGGGGCTAGTCGGGGCAGACGGGCCCACA
CATTGCGGAGCTTTTGACGTCACGTTCATGGCTTGTCTACCAAATATGGTCGTGATGGCG
CCGTCAGACGAGGCCGAGCTTTTTCACATGGTTGCAACCGCCGCCGCCATTGATGATAGG
CCTAGCTGCTTTCGCTACCCTAGAGGAAACGGGATCGGCGTAGATCTACCGCCGGGGAAC
AAAGGGGTTCCCCTTGAGGTAGGGAAAGGGCGAATACTGATTGAAGGGGAGCGAGTAGCG
CTCTTGGGATATGGAACGGCGGTTAGGAGCTGTTTGGCTGCGGCTTCTATAGTAAAACCT
CGCGGTTTAAATCTAACGGTTGTAGACGCTCGTTTTTGTAAACCGTTGGATCGTTCTCTC
ATTCGAAGCCTGGCGAAATCGCACGAGTTTCTTATTACAGTTGAAGAAGGATCAATTGGA
GGTTTTGGTTCTCATGTTGCTCATTTCTTGGCTCTAGATGGACTTCTTGATGGCAATTTA
AAGTGGAGACCACTAGTTCTTCCTGATCGATACATCGATCATGGCTCCCCAGCAGATCAA
TTAACCGAAGCAGAACAAATAGGAAAACAGAGTCATTGCTGTGATGAAAACAGAGGAATC
AGAGAGATTTCAAAGAACGGAAAGAGAAGGGCAGTTTAG
>EuDXS2
ATGGCGGTTCAGGGCTCTGTAATTGGACTCAACCAATCCTTCGCCCCATGTTTTCTCTCT
CCGAAGCTCAAACATTTCCCCAAAAAACAGTTCTTCGCCAGAGCTTCAGTATCCAAATCC
GACGGCGGAGATGAAGGGAAATTGATGATACGGAAGGAGAATGATGGATGGAAGATTGAT
TTCTCCGGCGAAAAGCCTCCGACGCCATTGTTGGACACAATCAACTTCCCAGTCCACATG
AAGAACCTCTCCGTACAGGATCTTGAGCAGCTAGCAGCAGAGCTAAGAGCAGATATAGTG
TATACAGTGGCAAAGATTGGTGGACATCTGAGTGCCAGTTTGGGAGTGGTGGATTTGGCT
GTGGCTCTTCATCATGTTTTCAATACACCTGACGACAAGATTATTTGGGACGTTGGACAC

CAGGCATATCCACACAAAATACTGACAGGGAGGAGGTCTAGAATGCACACAATAAGGAAA
ACTTCAGGGCTAGCAGGCTTTCCTAAAAGGGATGAGAGCACTTATGATGCTTTTGGAGCA
GGGCATAGTTCCACAAGCATATCAGCTGGTCTTGGTATGGCAATAGCAAGAGATCTTCTT
GGGAAGAACAACAATGTCATATCGGTGATCGGAGATGGAGCCATGACAGCAGGACAAGCA
TACGAGGCCTTGAACAATGCCGGGTTTCTCGATTCTAACCTTATTGTTGTATTGAATGAC
AATAAGCAAGTCTCTTTACCCACAGCTACAATAGACGGCCCTGCAACTCCTGTTGGAGCC
CTCAGCAGTGCATTAAGCAAACTCCAAGCGAGCCCCAAGTTCAGACAGCTTCGCGAAGCT
GCAAAAACTATCACAAAACAAATTGGGCCTCAAGCACATGAAGTAGCAGCGAAAGTAGAC
GAATATGCTAGAGGCATGTTTAGTGCTACAGGGTCTACTCTCTTTGAGGAGCTCGGTTTG
TATTACATTGGTCCAGTGGATGGGCATAACATCGAAGATTTGGTCACCATTTTCCAGAAA
GTAAAAGAGATGCCGGCACCGGGCCCTGTTCTTATTCACATTGTGACAGAGAAAGGGAAG
GGGTATCCTCCTGCAGAGTCTGCACCAGACAAGATGCACGGTGTTGTGAAGTTTGATCCG
GCAACAGGAAAGCAATTTAAGGTCAAAGCACCTACGCTTTCATACACACAGTACTTTGCT
GAATCCCTGATAAAAGAAGCCGAGGCAGACAGCAAGATTGTGGCCATACACGCTGCCATG
GGTGGTGGTACAGGCCTTAATTATTTCCAGAAAAGGTTTCCGGATCGCTGTTTCGACGTC
GGAATTGCAGAACAACATGCCGTTACATTTGCAGCTGGTTTGGCCACAGAAGGCCTCAAG
CCGTTCTGCGCCATCTACTCATCATTCCTTCAAAGGGGGTATGATCAGGTGGTGCACGAT
GTAGATCTTCAGAAATTACCGGTTCGATTTGCGATGGATCGCGCTGGTTTGGTTGGAGCG
GACGGGCCAACCCATTGTGGAGCATTTGATATTGCTTACATGGCTTGCTTACCAAACATG
GTGGTCATGGCTCCAGCTGATGAAGCCGAGCTGATGCATATGGTTGCCACAGCAGCAGTC
ATTGATGACAGACCGAGCTGCTTCAGGTTCCCAAGAGGAAACGGAGTTGGAGCAGTTCTT
CCTCTAAATAACAAAGGAACACCGATGGAGATTGGGAAGGGAAGAATTCTTATTGAAGGC
AGTAGAGTAGCGATTCTGGGATATGGTTCAATAGTTCAACAATGTCTTGGAGCTGCGGAC
TTGCTCAAAGATCGTAATGTATCAGTAACGGTAGCCGATGCAAGATTCTGCAAGCCTTTG
GATGCAGATCTAATCAAGAGATTGACCCATGAACATGAAATCTTATTAACGGTGGAGGAA
GGTTCTGTTGGTGGTTTCGGATCACACGTATCTCATTTTCTAAGCTTAAACGGTATTCTG
GATGGACCACTCAAGTTAAGAGCAATGGTGCTTCCAGATAGATACATTGACCATGGATCA
CCCCAAGATCAAATCGAAGAAGCAGGTCTATCATCCAGGCATATATGCGGAACAATACTA
TCACTACTGGGAAGGCCCAAAGAAGCTCTTCAATTGAAGTGA
>EuDXS3
ATGGGTGCTGCTTCTCCTCAGTACCCATTTGGTTTCGCCTCCAATTTCCATGGAAATTCG
AGGGTGTTTTCTCCCAAAGTGCAGTTTTCAGCTGTTAATTTCTCTTCCGGGGGGCAATTT
TCGCGAAGGGATTCCGGCCAAATTTCAGCCTCCACACTTAATTTCAAGGACCGCTTTGGT
ATACATTCTCTACCTGATGTTGGTGATGTATTTAGGGAGAATGCGCCAACACCCATGCTT
GATACCATCAAAAATCCCATGCATTTGAAGAATTTGTCTCCAAAGGAACTAAAACAATTA
GCGAATGACATCAGATCGGAATTATACCATACATTGTCAAAAATACAAAAACCGTTCAAA
GCTAGTTTGGCAGCGGTGGAGCTGACGATTGCAATACATCATGCATACGCGCATAAGATC
CTCACTGGGAGACGGCCCCTCATGCATACAATTGGACAAACGAATGGTCTTTCGGGTTAC
ACATCCCGTTTTGAAAGTGAATTTGATCCATTTGGTGCAGCGCATGGATGCAGTAGTGTT
TCTGCTGGACTTGGAATGGCGGTTGCACGAGATATCAAGGGGAAGCGGGAACGTGTAGTT
GCTGTCATCAGTAATGAAACAACAATGGCAGGTCAGATTTACGAGGCAATGAGTAATGCT
GGATATTTGGATTCGAACATGGTTGTGATATTAAACGACAGCCGCCATTCAATAAACCCA
AAACTTGAGGAGGCCACCAAAACCCCAATAAACGCACTTTCCAGTACTTTAAGCAAGCTG
CAGTCAAGTAAAATCTTCAGGAAATTCAGAGAAGTAGCTAAGGGATTTACTAAAAGAATC

GGTAAAGGCATGCATGAGTGGGCGGCCAAAATCGACGAATATGCACGTGATTCATTATCA
AATGATGGTCGGCCTCAAACGTATAGCGATTGTTTTGTCGAGTCATTGGTTATGGAGGCT
GAAAGAGACAAAGATATCGTGGTAGTTCATGCAGGAATGGGAATGGAACCGTCTCTTAAA
ATGTTTGAGAAGCGGTTTCCAGACCGGTTTTTTGATGTGGGGATGGCTGAGCAGCATGCT
GTGACATTCTCTGCCGGTTTGTCGTGTGGAGGACTGAAGCCGTTCTGTGTAATCCCGTCT
TCCTTTCTTCAGAGAGCTCTTGATCAGGTAGTTAATGACGTAGATCGACAAAAGATTGCG
GTGCGTTTTGTCATTACGAGCGCAGGGTTGGTTGGATCAGATGGATCAACCAACTGTGGG
GCATTTGACATAACATTCATGTCTTGTTTGCCAAACATGATTGTGATGGCGCCTTCCGAT
GAGGCTGAGCTCTCTCACATGGTGGCCACAGCTGCCCATGTCGATGATAGGCCCGTTTGC
TTCCGTTATCCTAGAGGAGCGGTTGCTGAGATCAACTTAATGACCGCCGGAGTCCCCATT
GAGATTGGCAAAGGGAGAGTCCTTATAGAAGGTAAAGACGTTGCTTTGCTTGGCTACGGC
GCCATGGTACAAAACTGTGTAAGAGCTCAATCCATTCTCTCAAACCTCGGCGTGGAGGTG
ACCGTTGCCGATGCGAGATTCTGCAAGCCGCTCGACGTCAGTCTCGTCAGGAAACTATGC
GAAGAACACGCCTTCTTGATCACGGTCGAGGAAGGGTCGCTTGGAGGGTTTGGGTCCCAC
GTTGCTCAATTCCTTTCACTTGATGGCCAACTCGACGGGAAGGTCAAGTGGAGACCGATT
GTTCTACCAGACAACTACATTGAGCACGCATCGCCGAAGGAGCAACTTGCTCTTGCAGGG
TTGAGCGGACATCACATTGCAGCAACGGCGTTAAGCCTTCTTGGTCGCACTCGTGAAGCC
TTACTCTTGATGTGCTAA
>EuDXS4
ATGGGTGCTGCTTCTCCTCAGTACCCATTTGGTTTCGCCTCCAATTTCCATGGAAATTCG
AGGGTGTTTTCTCCCAAAGTGCAGTTCTCAGCTGTTAATTTCTCTTCCGGGGGGCAATTT
TCGCGAAGGGATTCCGGCCAAATTTCAGCCTCCATACTTAATTTCAAGGAACGCTTTGGT
ATACATTCTCTACCTGATGTTGGCGATGTATTTAGGGAGAATGCGCCAACACCCATGCTT
GATACCATCGAAAATCCCATGCATTTGAAGAATTTGTCTCCAAAGGAACTAAAACAATTA
GCGAATGACATCAGATCGGAATTATACCATACATTGTCAAAAATACAAAAACCGTTCAAA
GCTAGTTTGGCAGCGGTGGAGCTGACGATTGCAATACATCATGCATATGCGCATAAGATC
CTCACTGGGAGACGGCCCCTCATGGATACAATTGGACAAACGAATGGTCTTTCGGGTTAC
ACATCCCGTTTTGAGAGTGAATTTGATCCATTTGGTGCAGCGCATGGATGCAGTAGTGTT
TCTGCTGGACTTGGAATGGCGGTTGCACGAGATATCAAGGGGAAGCGGGAACGTGTAGTT
GCTGTCATCAGCAATGAAACAACAATGGCAGGTCAGATTTACGAGGCAATGAGTAATGCT
GGATATTTGGATTCGAACATGGTTGTGATATTAAACGACAGCCGCCATTCAATAAACCCA
AAACTTGAGGAGGCCACCAAAACCCCAATAAACGCTCTTTCCAGTACTTTAAGCAAGCTG
CAGTCAAGTAAAATCTTCAGGAAATTCAGAGAAGTAGCTAAGGGATTTACTAAAAGAATC
GGTAAAGGCATGCATGAGTGGGCGGCCAAAATCGACGAATATGCACGTGATTCATTATCA
AATGATGGTCGGCCTCAAACGTATAGCGATTGTTTTGTCGAGTCATTGGTTATGGAGGCT
GAAAGAGACAAAGATATCGTGGTAGTTCATGCAGGAATGGGAATGGAACCGTCTCTTAAA
ATGTTTGAGAAGCGGTTTCCAGACCGGTTTTTTGATGTGGGGATGGCTGAGCAGCATGCT
GTGACATTCTCTGCCGGTTTGTCGTGTGGAGGACTGAAGCCGTTCTGTGTAATCCCGTCT
TCCTTTCTTCAGAGAGCTCTTGATCAGGTAGTTAATGACGTAGATCGACAAAAGATTGCG
GTGCGTTTTGTCATTACGAGCGCAGGGTTGGTTGGATCAGATGGATCAACCAACTGTGGG
GCATTTGACATAACATTCATGTCTTGTTTGCCAAACATGATTGTGATGGCGCCTTCCGAT
GAGGCTGAGCTCTCTCACATGGTGGCCACAGCTGCCCATGTCGATGATAGGCCCGTTTGC
TTCCGTTATCCTAGAGGAGCGGTTGCTGAGATCAACTTAATGACCGCCGGAGTCCCCATT
GAGATTGGCAAAGGGAGAGTCCTTATAGAAGGTAAAGACGTTGCTTTGCTTGGCTACGGC

GCCATGGTACAAAACTGTGTAAGAGCTCAATCCATTCTTTCAAACCTCGGCGTGGAGGTG
ACCGTTGCTGATGCGAGATTCTGCAAGCCGCTCGACGTCGGTCTCGTCAGGAAATTATGC
GAAGAACACGCCTTCTTGATCACGGTCGAGGAAGGGTCGCTTGGAGGGTTTGGGTCCCAC
GTTGCTCAATTCCTTTCACTTGATGGCCAACTCGACGGGAAGGTCAAGTGGAGACCGATT
GTTCTACCAGACAACTACATTGAGCACGCATCGCCGAAGGAGCAACTTGCTCTTGCAGGG
TTGAGCGGACATCACATTGCAGCAACGGCGTTAAGCCTTCTTGGTCGCACTCGTGAAGCC
TTACTCTTGATGTGCTAA
