# Supplementary material for: Genome-Wide Identification and Expression Pattern Analysis of the DXS Gene Family in Eucommia ulmoides
Source: Plants (Basel). 2026 Sep 17;15(18):2843. doi: 10.3390/plants15182843 (PMC13610960; doi:10.3390/plants15182843)
Supplement: Supplementary file 1 [file plants-15-02843-s001.zip › File S2 The genomic sequences of four EuDXS genes.pdf]

>*EuDXS1* EU0120438.1 CAFEUEU0120438 Chr11:41015600-41026590(-)
ATGGCGCTCTGTACATATTCTTCATTCCCTGGACATTTTAACAGAGCTGTGGTGGCTTCA
GATCTCCAAAAGCAATTTGGTTTTTGTTCCAAGGGGAAAGATGTTCACCAAAAACATACC
CAGgtttcaattctctctcactctctctctaattaagaaatggaagatggattcaagaat
gctctgttactgaacatggaaacagaaacagaaacagaacagaacagagcatggttttgt
tggtttgatttgtcctgttctttaactcttttttttttttttttttagtggctaataa
tttcttggaggatgaacccaatttatgaacccaattttttttttgctgaaatgtgtgtt
tttttgttttgttctgatttacagCTCAAGAAAAGATCAATTGGGGTCTGGGCATCTCTA
TCGGAAACAGGGGAATATCACTCCAAAAGACCTCCAACCCCACTTTTGGACACTATCAAC
TATCCAATTCATATGAAGAATCTCTCCATCAAGgtatgtaaaaaattgaaatcacacaca
cacgtgtgtgtatatatatatatattgttttacgtgtgcctatttaagctaatcagctct
ccatctccatacactcttatcttaaaaatactctcacatcggtttgatatagaaataaat
taaaaaatttaattcctagagtggttttcaagagagttaaactatgatcgacaagaaaag
agagcatccttgtcaacctgactaacatctctttagcgtgagcgcgcacacacacatata
tattaataaatgaggatgttttctttgaaaatgtattttcgtttgagacttggattcatt
tgaaggtaatggatgattgagatggattgagagtttgttatttaatttctcaacaaaata
tgaatcaacaattggaaattgttttctagggatgtcactagttcggttttggtctggttt
catataaagttaaaaccaaaatcactagaaatatatcaaaccaaaaccaaaaccgtcagt
tcggttttaaaatcaaatcatttaaatttacacaaaatcaaaaccaaaatcgcgaaacca
tttaagatttttttaattttataaaaaccaaaaccgcaaatcgttgtttttttatatataaa
taaaaacataactaaaatatcaaatataaaatattaattgattttattatacttgttagg
gcactattaattaacctttaatttcaattttaatctctcttttttttaaatttattttat
tatatctattaggacacaattaattgacctataattaaattattcaattacaatatgaca
taaaattgtgccgtaatataatttttatttttagggcactatttgttgtctctttatatt
atatatagaatattaatttttttttcactaatttaattttatcgggacactattttttacc
ctttaaacatttgattatatattagttgttaataaaataataagaaaatcaatattaaaa
atataaaatttatgcgacactttcaattttgtaccttgaaaaagcacctttatagggcac
attacatatagtatcctaataaaagtgccttaatagacaatttttgttgtagtaaaaatg
gctaaaagtataaataaataaatatatacatatattaaggttaaaatgtaaatatattta
aaaaataaagggtatatatgtattaaaatatttttaaattatcgattccatggtttcgca
gtttggttcatggttcggtttgcagttcggtttcacagtttttatcccaaaaccaaaacc
aaaatcgtttcggttgctaagttcaaaaccgcaaccgcaaccaaatcatttaatcagttt
taaaaccagaccattcagttcgctttctctcggttatccatcagttcggttttttgtgat
atccctattgttttccccttaaaatggtcaaaaatattttattgggtagtttgtagggag
ttccacgagctaaatcggtggttgtttatttcttgggagttccatgatctcaactcttag
atctaatccgaaggctgaaaatgtcccataaaattctttaattttgacctttgaatatgt
aatccaagggctgaaaatatggattataattcaatatatttagttgagtttttttttgcac
tttgaacctctgaagtttcagaaaaatctcactttatcccttgttctttcaatttgaaca
tattaaaccctgaattattcaaatcgtatcaagtcaatcatttcgtctttttttttacga
aattcacccacgtgggtgattccatgtttgacacgtgattgatttttgtcataaaaataa
tagaattatcgatttggactgacttgaatgatttggatactttaggtttaatatgttgca
aattgaacgtacacgtctcaaatgtaagatatatcctgattaatatctatagaatagata
catacatacatatatattagtttatataatttaatatagaattttaaattcagGAACTGA
AACAACTCGCTGAGGAGGTGCGATCTGATGTGATCTTCAATGTTTCCAAGACCTGGCGGT

CACCTCGgttcgagccttggcgtggtcgagttaaccgtggctcttcattatgtgttcaat
gctcctaaagatagAATTCTCTGGGATGTTGGTCATCAGgtgataatttttttttcgat
ccaaaattgaataagaaaagagtgaaaataaggacccgtttggttgtcgttcaattcgca
aattgatttaaaaataaaaataaaaaattagtcgatttatgaattgttatagttcaaggt
tcttttgtgcaaatttggaaactgttagaatggaataatttatagacaaccagacgggac
caaaaatgttgttcaaataagtgtttctgtttttgtttttctaattttgttctcaattta
tcgaaaaaaaattggaaaaaaaaatgtgttatgttctatctccggattattagaacaaaa
ttttgggattagaacatataaaaaatagattgtttttcttgtttgtgttccaactattt
agggaacaaaaacaaaaataataaaagaaacgcggcctaatggattttatgtttatcgaa
ttgagttgtgtagTCTTACCCACACAAGATTTTGACTGGGAGAAGAGACAAAATGCATAC
GATTAGACAGACGAACGGGCTTGCTGGATTCACTAAGCGCTCTGAGAGCGAGTATGATTG
CTTCGGCACAGGTCACAGTTCTACCACAATCTCAGCAGGCTTAGgtgagattgatttgaa
tttgggaacatttttcttttctattcccataatgttgttctaaaaatcttggatcaaaac
aaatatatatatatgtgcgcgcgcatgcggctagtacttaaaaaaacgttacctgaatta
ttttctcatttttcgacagGGATGGCTGTGGGAAGGGATCTAAAGCGAAGAAAGAACAAT
GTAGTTTCCGTGATAGGCGACGGTGCAATGACGGCAGGTCAGGCTTATGAAGCCATGAAC
AATGCCGGCTACCTCGATTCAGACATGATTGTCATCCTTAACGACAACAAACAGGTTTCA
TTACCAACCGCCAACCTCGATGGGCCCATTCCACCCGTCGGAGCTTTGAGCTCTGCTCTC
AGTAGATTGCAGTCCAACCGGCCTCTCCGTGAACTTAGAGAAGTAGCCAAGgttcgtatt
ctttactaggcaaattgtgtcaaaccacgttatatcttgtgtcacttttgtatttgttat
tttctctattttcatattttttgtcgtctccgaacccaacatttttttcatattattttg
gattgttgcagGGAGTTACGAAGCAAATCGGGTGGCCTATGCACGATCTAGCATCGAAAG
TCGACGAATATGCTCGCGGCCTAATAAGCGGTTCTGGATCGACTCTATTTGAAGAGCTTG
GACTTTATTATATCGGTCCTGTAGACGGTCACAGCATAGATGATCTTGTTGCAATTCTTA
AGGAAGTCAAGAGTACTCGAACAACCGGCCCTGTTTTAATCCATGTTGTCACCGAGAAAG
GAAGAGGATACCCTTACGCTGAGAAAGCCGCCGACAAGTACCACGgtaaaaattcgtgtt
ccacggtagcgcgtggaatgcataggttaaaattttaatttcatgaacggttcaaaaact
agttcctttaaacgaccaatttttggatcagctgcttacaaaaaaaaaaaaaaaaaaa
aaaacaacaacgatgataaaaaattataagcgacttatttcaaagataatctaatacagG
AGTGGCCAAGTTTGATCCGGCTACAGGACAGCAATTCAAAGCTAGTGTCAAGACTCAATC
TTTCACAACATATTTTGCCGAGGCTTTGATTGCTGAGGCAGAAGCCGATAAAGATGTAGT
TGCGATTCACGCGGCAATGGGAGGAGGAACCGGTTTGAATCTCTTCCTACGTAGATTCCC
AACTCGGTGTTTCGACGTGGGGATAGCCGAACAACACGCGGTCACTTTTGCCGCCGGTTT
GGCCTGTGAAGGACTAAAACCCTTCTGCGCTATTTATTCTTCTTTCATGCAGAGAGCTTA
TGATCAGgtattcccttcttagtgcccggtttggcggaatatctaaaccccactgtgatt
agatcaaattgtacttaagtcttaagcatgggatgtaactggtgacaaaaatatcataga
ggtgcaaagtacaattcggtctaattagaggaagatatattaaaacccccccccaaaaaa
aaagttggctttttgtttttaacagaatttttgttcccagatttctgtttttgttccatg
tttttgggtgaaatcgatggttttgaaatctcaaaactaaggccctgtttgttttaact
gtaatatgggagggaaaattttgtttttgttttgatcccagaaacaaatttctgtggata
taataaaattgttcttatttttttgctcctaaatggaacataatagaatgtttcctatttc
agttcctagtttttttgggaacggaaacactaacaaactgccacctaagacgtcgtttgtt
tgtttttcttggaacggaaatagaaaatgaaaaaaaaataaaaaaaaaatcacttccatgtt
cgagtttttttttgggaacataaacactaaccaaacgtgacctaattgattttgcacttct

tattgaaatgttattacaagaaattactaatctaaaaaaaggttatctgtggccttagga
ggcatgtgtttagagtttttgttcccaaaaaacgaggaacagaaacgaaaatatgaaggg
aaaaaaatcattttctgttacatttccatatttttagaacaaatttttgggaacataaca
gaattacaagaaattacacttactaaaagaacactaaccaaacacgacctaaacggtatt
tggcacatggtaaatatatagGTCGTGCACGACGTCGACTTGCAAAAGCTTCCGGTAAGA
TTTGCAATGGACCGGGCGGGGCTAGTCGGGGCAGACGGGCCCACACATTGCGGAGCTTTT
GACGTCACGTTCATGGCTTGTCTACCAAATATGGTCGTGATGGCGCCGTCAGACGAGGCC
GAGCTTTTTCACATGGTTGCAACCGCCGCCGCCATTGATGATAGGCCTAGCTGCTTTCGC
TACCCTAGAGGAAACGGGATCGGCGTAGATCTACCGCCGGGGAACAAAGGGGTTCCCCTT
GAGgtatgaattaaattattctcctggttgcttaagtagtgtttgtttttgggattccg
ttaaacttaacggtcaaatcttaaaaaatcaaaattaataaattaattaatataaattga
cactaatatccccgagttgggttcgatcagataatatccgtttgcttttttcctttcct
ttattcttccggtttgggtcgtgttggatataagggtaattttgtcatttttgtgaaaat
accacaggagtgtaaagtacaattcgatcaaaccacatgaggacaaagtgccattaacga
aaaaaaaaattattcgagaaaaaaagtgttaccaaatggtgctttattttttttctata
tttagGTAGGGAAAGGGCGAATACTGATTGAAGGGGAGCGAGTAGCGCTCTTGGGATATG
GAACGGCGGTTAGGAGCTGTTTGGCTGCGGCTTCTATAGTAAAACCTCGCGGTTTAAATC
TAACGGTTGTAGACGCTCGTTTTTGTAAACCGTTGGATCGTTCTCTCATTCGAAGCCTGG
CGAAATCGCACGAGTTTCTTATTACAGTTGAAGAAGGATCAATTGGAGGTTTTGGTTCTC
ATGTTGCTCATTTCTTGGCTCTAGATGGACTTCTTGATGGCAATTTAAAGgtaatatata
tatatgtgtgtgtgtgttcattaaatgaaaaattttaaattttgttttggacataaaaaa
gaaattttaaacattaaggttcattgtttttccattgaaattgagaacggaaatagaat
tgtcgaaacgaaaaaaaaaaagaattttggaacaagtttttgggaacagaatggatttgt
tccatattttgaaaaagtaatttatgatttttttgggtctctgtaaatgatcttttaat
tcaaaaaaaaaaatatagataaatgaaaaaaaaaaaaggggatttttccgtgtaaatttg
ttcttcagtgtaaaacaaacgatatttttaaaattaaatatttttttccttaaaaaacaa
agaaatacttaaaaaataaaattaccaaacagtgccttaattttatttttattttttta
cagTGGAGACCACTAGTTCTTCCTGATCGATACATCGATCATGGCTCCCCAGCAGATCAA
TTAACCGAAGCAGgtcttacaccgtctcatatagccgccacagtattcaatatactcgga
aaaacaagagaagctcttgagatcatgtcataatagAACAAATgtaagaagatcttaaat
ttttcatcttcctatgaatgtacaaaagttaaggccgcgtttggtttcacaaagttggag
cctcggaattgcaattgaaaataaacgcattgaaaaaaaaaaattaaaaaaatcatgtgc
ttatttctaattccaattccgacgttgcgattccatgcttccaaacactaagatagtgtt
tggtcgtatggaatttgagcttggaattggaattgaaataaatgcatataaacttttaga
tttttacatgcgtttttttctaattccgattctgaggttgtaattacatgcaaccaaacg
cgaccttagtgttttctgttctagcttaaaagataaatagaaatcaatagcagtaaatat
gtgtttttagcaatatattgttcagtgtaagaaaaataagggtcagtttgggttttaag
acacattgtttggatacctaacctatgttttagatgccctaaaaacctaaaatcctaaaa
tcctagttgtctgtgccaaacaggtccttaacaattgtttagcacatgtactttgaaggg
gtttttgtcaacagtccaaaaaattaaaattttagctcacgcagttcacgtgctataac
aatttttcaaaaagtctaaaattactgtaaaaaatcgaaaagtcattgtacgctataaaa
ttaagagagatttttgagatgtcccaagattccaaaaatagtttttaagaaccatttc
atgtgcaaaaaaatcactgttatcggactccataaagtgctatttttatagaatgggacc
tttgggcaaattttcctaaaatttactgcaatttttacatggaattatagtaattttgg

acttttagaaaattactgtagcgcgtggattgcatgggttaaaattttaattttatggat
tgttaactaaaaatccctactgtagatttctaaaaccgaaatttagagtattcaaacatg
gtgccaaacgggtccttaactgttctgttttatcgaagtaatgatttagtttaagggaaa
ataagggcctatttggttttcaattttcttaaaaagaacatttttatgcaaatagagatt
cattaagacattgtttggatatataccttaacacgatttttgaaaaacccaatttaagg
tctttgaagatggtgccaatcaggtccttaaccgtagggaaaatcatatttttggttctt
atagtaaggggcgggtgtttatgtagaccctaaggtttgtcaaattgcacatttagactg
tcaattttcatatttgtttcgaccccgcaaaattgataacatttttaatgattttggacg
gaaaattgattgtactcagcacatggccttctaatgggaatattttcgttattttataac
ttcattaaccgtaaaaagtttttttttaacattttcttaataacgattcatctgaaaaat
aacagagaaaaacatgaatttttgttaggggataacgtcttaaatttagtggagtgatga
aataacgaaaaaaatattcccattagaaagttacgtgctgagtgcaacccattgtccatc
tgaaattattaacaacattttcaatattgcgggattgaaacaaatgtggaacttaaggtg
gtgtttggttagtgtttctgtttttctttttctatctctaataagttgggacgataattt
tatttccgttctggagtattacagaaatagaaatttttttggttacgaccattgtcctga
aattgcttcttgtaatttacgagaccagaaacaaataaacatgtttggttgcaacatttt
tttttaaaatatgtggtgatcggcgaggtggtggtggccttggctgacagggtgataatg
agaatagttttataagagtgaatagaaatcaatatttctttttttgtttatcaaattctta
aggaattcaggaattactttcaaattattctcaaaattttggaaacagaaacccattcac
caaacatgtttctatacattaatcccaaaatttgggagcgggaccaaaaatcaaggaacg
aaaacagtaaccaaacggggcctaagagatcaaatgtacactttggcaaatctcatggtt
cacattatagggaccgaaaatataattttccctaaccgtacaactttttaaaacaagggg
aacaaaacgaaacaaaatcaaagtatgaggggaacaaaatgtaattaaacaaagaatttt
cacatcactgaacatgcatttggattctcctcatcaaacatggtgcataacttactgcct
ccaaaaaattcaataaccagaggatcatcgctaaggccctgtttggctgtttctggattt
tctttttccttgttttcatctttttcttgatcttctttgttgtcaatcacgatttcgact
ttcttaccagtcttcttccttaatttcttcaaaattttgtctgggttgatcttacccttc
acttttactctgtgtttgttcatgtccgttgtaaatgtctccacccccttgcaaatttcaa
aaattggaaattattgtgtatggatttaataaataaaaacagaacaatgtatacatatat
agagagaggggctagagagagacaggagctgataatcggattatgagtgtgagggaggct
cacattatgagctaactttcagggttaaacgaggtccaagaccaccctatactggaatca
tagatttataacctaggtcacaacaactctggctcaataactacgaaggaagagattggt
tgaaagttaactgaccaaagacccaatatgtgacggggaaattattgggtttatcttata
ttggatgtaaaaatagttggtggtcaggttacaaatgtgaggaaaaaactcatctcatta
gttagcttttgcggttgagaaagactcaaaatcaacctacactgatattaaagtctatgt
tacaataattatgacctaataactacgaaaggaaaattagttgagggttcgccgactaaa
tgccagatgtgtgagggtgagattgttggatttatctcacattagatgtgtaaaaaattg
gtggtcaagttataagtgtgcagaaaaaacttgcctcattaattagtttttggggttggg
aaatatccaagaccatcttatactgatatcagagtccaagactccacgttacaactatgc
ccaataactacaaaaaggggagattgattgaggattaatcgatcaaaaatccgatatgtg
aggggaagattgttaattttattctacatcagatgcgaaaaggcctggtgatcaggttat
taatacaagggaaagactcaaagttagtttttgaagacgagagaagaccgagaccacgag
gtagagagaggaagagaaagagagagagggaaccttggcaacagttctctcacaagcata
tatatatatatatatatataaacatatagagagagagaggaaaccttggattttagaa

atggccttggcaacagttctcccaaaaacattacaatacatatatatcgatatatatata
gagagagagagagaggggcagagagagaaaaggaaagagggtagagagagagagaggaaa
cctttgattttggaaatcaccttggcaacagttctctcacaagcattacaatacatcgaa
acattgaattccgccaccataacctgagaatcgacccaagaaatcaatcaaataacatcg
atttaatgcacaattcgtagaacccaaatcgcaatttacgcaaatttatcaattttgtgt
ataaaataaatatacacacacatatatgtatatatatattgtatgtatgtatgtatcgtt
tcttactttgtctttttgattttcttttcatccgccattgaaattttacagAGGAAAAC
AGAGTCATTGCTGTGATGAAAACAGAGGAATCAGAGAGATTTCAAAGAACGGAAAGAGAA
GGGCAGTTTAG
>*EuDXS2* EU0127987.1 CAFEUEU0127987 Chr16:24443555-24449151(+)
ATGGCGGTTCAGGGCTCTGTAATTGGACTCAACCAATCCTTCGCCCCATGTTTTCTCTCT
CCGAAGCTCAAACATTTCCCCAAAAAACAGgtaataattcaaaaaactcttctcattcgt
acatcccccattaacctctctctttctctctctgaatctggatgttgttgatcatgtt
ctacagTTCTTCGCCAGAGCTTCAGTATCCAAATCCGACGGCGGAGATGAAGGGAAATTG
ATGATACGGAAGGAGAATGATGGATGGAAGATTGATTTCTCCGGCGAAAAGCCTCCGACG
CCATTGTTGGACACAATCAACTTCCCAGTCCACATGAAGAACCTCTCCGTACAGgtctgg
tagtggtacacgctcaccacacatgatgatagccccccaccacgaccgactttttttttt
ttttttttccctggagtatgagttacatgccacgtgtctgtttttgatttgacagGATCT
TGAGCAGCTAGCAGCAGAGCTAAGAGCAGATATAGTGTATACAGTGGCAAAGATTGGTGG
ACATCTGAGTGCCAGTTTGGGAGTGGTGGATTTGGCTGTGGCTCTTCATCATGTTTTCAA
TACACCTGACGACAAGATTATTTGGGACGTTGGACACCAGgtgaaaaatcacctccacta
acctaactctgttttttttttttttttttttcctttcttaaattatacggtatcgtatgt
tcggaccagtttacttacttcaattaatcatctcgctaggcgatattgagggcgtactct
cctatattgaccttgtgcattcatgattaaatttaaaatttaattcctgaaaaagttgaa
ctttaattgacatacctaaaccgggtgtgtctggactagtttacgtatacttcgattaat
catcacactaggtgacattaaaagtgcaatctcccatatcgttctgatacatttaggatt
aaattcaaaatttatttcttgaattgtccctgcaaaagttgaactttaattaacatacct
caattggacacaccggttttttttttcccaaaattaaaaagagaaagaaacagtattagc
atatataatctgttcggtacttgacagatccaactgttgtggtctattaaattcaaaatg
atgtgccaagcaaaagctggatttagtgctactttttttcaaactgtaaatttctgattta
aaagcatgggcagagccacaaatttgggataaaggggcagaaaattttagagggataatc
tgatatcttttgctggattcttctcccgacaagtcaaaagaggaataattggtgaagcat
ggataatatgttgttagttcctttgaaaacataaattttgatttagtctcacgttttaaa
ttttatttgcgtattaatcccacatgcactcacgcattgcgcgagatgtagaaagaattt
gatgagtaaattcttattataaagaatattagttacatctaactaaaattagttacaatt
taataattaactatattatatatatatctgtgtactgtacctgtgtaatattagttacac
gttacaaattacatgtatttgtgtactatttctgacatctaacaaatattacacataaat
tagtttaatattaatttcatgtatttgtgtaatatttgttacatctaacaaatttttgca
tacaaattaattacatattacaaattatagtaatatttgttacatctaacaagtatttta
tgtacaaactaataaatgtaattgcgcgagtatagacgcgtttattgagtcttccgtctt
ttttaatacgtgggactaaaatacaagttgcaattgtttatacgactttttaaaaattaa
gggtaaactaaggaacttaccatcgaaacaccctcaaaagagttgctccacttgaaatta
tatggttgtaagtttgaacttcctaatatggtatcagagtaaaatttgtctctcattccc
ttatactaatcacccgtgcaaactggggtttgcagatttctcataatttcctcgtaatca

aatcatccgtgcaaactagtggttacatttgatggcaggtgttacattcatatcccacat
caatcatttgagagttcctaatccactatataaattaaaagagttatttcaattaaaatc
atatagttttaagttagaacctcctaacaatattaatataaatatgggtatataagtata
aaaacaaaaaaaaatgaagggggtcagaggccctcctgggccttcatcggctgtccccatt
taaaagtgccggtgttaggagcaatttgaaaggcaaatagatgtttacatgtgtagttca
aattctttgtcaaaatcaattcaaattgggtgtttgtcattgcactccaactgcagtgcc
gaactggcccttagtttgtgattgcaaaaatgatttatttatttttatttttgaagtat
atacatagaaatggaaagatctgagtaaaacaataattgttgcagGCATATCCACACAAA
ATACTGACAGGGAGGAGGTCTAGAATGCACACAATAAGGAAAACTTCAGGGCTAGCAGGC
TTTCCTAAAAGGGATGAGAGCACTTATGATGCTTTTGGAGCAGGGCATAGTTCCACAAGC
ATATCAGCTGGTCTTGgtatgagcaattctctccccatctcatttttttttttttttaaa
ttgtgaaaaaaaaatatacttttactaaaaattgagcttttttttttgtagGTATGGCAAT
AGCAAGAGATCTTCTTGGGAAGAACAACAATGTCATATCGGTGATCGGAGATGGAGCCAT
GACAGCAGGACAAGCATACGAGGCCTTGAACAATGCCGGGTTTCTCGATTCTAACCTTAT
TGTTGTATTGAATGACAATAAGCAAGTCTCTTTACCCACAGCTACAATAGACGGCCCTGC
AACTCCTGTTGGAGCCCTCAGCAGTGCATTAAGCAAACTCCAAGCGAGCCCCAAGTTCAG
ACAGCTTCGCGAAGCTGCAAAAgtacgtaaatatatgtattataaaaaaaagtctctttct
tccctaaggcagcatttggtagtggagaattcaagtctcgaaattggaattggacaaaaa
tgcatgtaaagttataaattggacaaaaatgcatgtaaagttataagtttttacaagcat
acgtttctaatttcaattctgaggctccaattacgtgaaaccaaacactacctatctttc
gtctataatttcagtgagatctgataagctttttttttttgctttctaatttgccgaaa
agACTATCACAAAACAAATTGGGCCTCAAGCACATGAAGTAGCAGCGAAAGTAGACGAAT
ATGCTAGAGGCATGTTTAGTGCTACAGGGTCTACTCTCTTTGAGGAGCTCGGTTTGTATT
ACATTGGTCCAGTGGATGGGCATAACATCGAAGATTTGGTCACCATTTTCCAGAAAGTAA
AAGAGATGCCGGCACCGGGCCCTGTTCTTATTCACATTGTGACAGAGAAAGGGAAGGGGT
ATCCTCCTGCAGAGTCTGCACCAGACAAGATGCACGgtgagacagaaactgtttttgttc
tgtttaaacaatccagatttttacatcaaattccatggaacagaacacaactgttccca
gatttcagtttctattctaagtttcatgggaaaacactaggaacaaaagagaattatccc
ccagtttttgtttctatttcaagctttttgggaactaaagcacttaacagattcagctta
tttagtgattctgttctccgatttctgttttctttccaagaaaacttgaaccataaacag
aaatattggggaaagtcagtttctgttctgttcccagatttttagaacaaatttcaggga
accaaacaggatcttccccatatttatattttgttttcaatttttttgcaatcagaaata
gaaagcgagaacaaaaacactaggcaaacaccacctatataaacgaggccaaaacaaacg
cgacccaccttggttttctcaatttatttggtagaaaaattcttactttgttgtgtttcc
agGTGTTGTGAAGTTTGATCCGGCAACAGGAAAGCAATTTAAGGTCAAAGCACCTACGCT
TTCATACACACAGTACTTTGCTGAATCCCTGATAAAAGAAGCCGAGGCAGACAGCAAGAT
TGTGGCCATACACGCTGCCATGGGTGGTGGTACAGGCCTTAATTATTTCCAGAAAAGGTT
TCCGGATCGCTGTTTCGACGTCGGAATTGCAGAACAACATGCCGTTACATTTGCAGCTGG
TTTGGCCACAGAAGGCCTCAAGCCGTTCTGCGCCATCTACTCATCATTCCTTCAAAGGGG
GTATGATCAGgtgaggatgaaaaattggaaccgaaacagaaaactgacagcaaaaaaataa
aataaaattgtttttgttctggtccacgatttttcagaacaaatttctggaaacagaatg
aaattgttcatagatgtctattcatgttccgagaattctgggaacaacaacactaaacaa
atgccgcctatggccatttagggtttattaacacattttatgatctatatgaagGTGGTG
CACGATGTAGATCTTCAGAAATTACCGGTTCGATTTGCGATGGATCGCGCTGGTTTGGTT

GGAGCGGACGGGCCAACCCATTGTGGAGCATTTGATATTGCTTACATGGCTTGCTTACCA
AACATGGTGGTCATGGCTCCAGCTGATGAAGCCGAGCTGATGCATATGGTTGCCACAGCA
GCAGTCATTGATGACAGACCGAGCTGCTTCAGGTTCCCAAGAGGAAACGGAGTTGGAGCA
GTTCTTCCTCTAAATAACAAAGGAACACCGATGGAGgttagcatcattaacaattatctt
ggaaaaatagtaagaaaatgaaaaggtcaaattaattttttttccccgaatatttaaca
gATTGGGAAGGGAAGAATTCTTATTGAAGGCAGTAGAGTAGCGATTCTGGGATATGGTTC
AATAGTTCAACAATGTCTTGGAGCTGCGGACTTGCTCAAAGATCGTAATGTATCAGTAAC
GGTAGCCGATGCAAGATTCTGCAAGCCTTTGGATGCAGATCTAATCAAGAGATTGACCCA
TGAACATGAAATCTTATTAACGGTGGAGGAAGGTTCTGTTGGTGGTTTCGGATCACACGT
ATCTCATTTTCTAAGCTTAAACGGTATTCTGGATGGACCACTCAAGgtaacttttcataa
gtcttttacgtccaattttcagaacaaatacgtgggaacagaacagaattgttcctacgt
tgctgtttccgttctttctgggaacagaaacgctaaacgaatgtcaccttagtttcgtct
tattgttcgaatctccacatgaaactaactctgtttaaaactttcaccagTTAAGAGCAA
TGGTGCTTCCAGATAGATACATTGACCATGGATCACCCCAAGATCAAATCGAAGAAGCAG
GTCTATCATCCAGGCATATATGCGGAACAATACTATCACTACTGGGAAGGCCCAAAGAAG
CTCTTCAATTGAAGTGA
>*EuDXS3* EU0108829.1 CAFEUEU0108829 Chr05:1720404-1729474(+)
ATGGGTGCTGCTTCTCCTCAGTACCCATTTGGTTTCGCCTCCAATTTCCATGGAAATTCG
AGGGTGTTTTCTCCCAAAGTGCAGTTTTCAGCTGTTAATTTCTCTTCCGGGGGGCAATTT
TCGCGAAGGGATTCCGGCCAAATTTCAGCCTCCACACTTAATTTCAAGgtacgatgcttt
gattttccttaggtagcgtttgggagaacgaaattgaaaccatttggaaatggaatttga
aatgaacgcatactatcaattcaaattcaaattccatgcaaccaaacactaccttatagt
ttttccccttatagaaattgttagattcctgtcccaatatcgagcacttgagagttctc
ggttaccaccatatggttttaagaacaactggtattagaggcaaggttataagaatttgc
ctcacatttgtacattttctaatggccaaattatccgtgcaagttggggttgcaaattgt
tcatcattccctcgtaatcaaatcatccgtacaaacttggggttacattgaatggcggat
gctagattcctgtcccacattgagttcccaaactactatataaatcaaaagagctactcc
aattgacactatatggttttaggttggaacctcttaatagaaattaagaattgtattgag
ttggggttgcaaattgttcatcattccctcgtaatcaaatcatccgtacaaacttggagt
tacattgaatggcggatgctagattcctgtcccacattgagttcccaaactactatataa
atcaaaagagctactccaattgacactatatggttttaggttggaacctcttaatagaaa
ttaagaattgtattgagttggggttgcaaattgttcatcattccctcgtaatcaaatcat
ccgtacaaacttggggttacattgaatggcggatgctagattcctgtcccacattgagtt
cccaaactactatataaatcgaaagagctactccaattgacactatatggttttaggttg
gaacctcttaatagaaattaagaattgtattgagggtaaaatttgatatttagtttctct
acaaaatctaaatcaacaattgaaaatgtctctccagctgtccaattgtcaaaaatattt
aaaatataaaatattcctgatttattatatagataatatagattttaatccaatacaata
attttgaacgtcctatagttagttcaaatacattaatcatgaaaagacattttgttcgag
agattgatatttggtttctcaacaaaatctaaatcatcggttgcaaatgtctccaaccat
ctaatgataaaaaatctctgaaatgtcgaatatgtcttgatttattatatagattataca
tattttaattcaatacattaacctcagttagtgtttttcttttgcaactcctgatgtagG
ACCGCTTTGGTATACATTCTCTACCTGATGTTGGTGATGTATTTAGGGAGAATGCGCCAA
CACCCATGCTTGATACCATCAAAAATCCCATGCATTTGAAGAATTTGTCTCCAAAGgtta
agaaatcggcgttctttatttacatatttttgctgttttaagcccgaggcttttatttta

aatccgatactaagttgattgcttgtgtgcagGAACTAAAACAATTAGCGAATGACATCA
GATCGGAATTATACCATACATTGTCAAAAATACAAAAACCGTTCAAAGCTAGTTTGGCAG
CGGTGGAGCTGACGATTGCAATACATCATgtttttcaagctccattggacaagatattgt
gggacatgggggaacaagtacatatatatgtgtatatatatacatacatataattatata
tgtttatttattataaattaggacggtgtgggcttcatataataattggtgtcaatagaa
tggatgagatctgttagtgaaaatgtggatttaatctcaaccattcacctgaagaaatat
tttcatcctttggaccatatatttgaagagattctagtctttctatcgaatataattact
ttgtagagcacacaccattctaatttataatacaaaatttattattttttggaatttgtt
agttgaaatttgaaatttgaaattacgtaaatttttttattgaaataatattaatgagtgc
ctttaggtacaagcacttactagtgtgtttatatatattttgtctttgatgtatgcagGC
ATACGCGCATAAGATCCTCACTGGGAGACGGCCCCTCATGCATACAATTGGACAAACGAA
TGGTCTTTCGGGTTACACATCCCGTTTTGAAAGTGAATTTGATCCATTTGGTGCAGCGCA
TGGATGCAGTAGTGTTTCTGCTGGACTTGgtatgtgtgtgtgtatatatacaacaaaaac
aaccaaggctttgtcccactacaatatatatatgtgtgtgtgtatacatatatattggtg
tgtgtttttttaaatttttaacaagtgtgttttgaaatagtctaaagtctaatccaatctg
ctttgtttgtctgtgttttatagatattcaatgatgcctccaaattcaattctcgacaaa
aaacaaaaagagggttaatggcactttacctcgttgtggtttgaccgaattgcacttatc
ccccttggtattttcgtaagcacttacccatctgtggtttagattaaagtgcaatggtca
cattttccgttgaagttaatggtcaaacctcaaacgtcaaaattaataaattatttaata
tcaattgacatttataccaccagatcgggtcgagtaggataatatccatttaccttctgt
ttttttcaccaggtaggatcgggttcaatataagggtaaattttctatgtcacgcaattc
tattagttatctaaccgttgcactttagttggggccacggggggtaagtgcttatgaata
tactacagcgaggtaaagcgcaatttggtcaaaccaggggtaaagtgcgttaaccccaaa
actaaataaatcattaacagctctatctctatgctatttgagtataaaaacttcaaattt
tgagcatgatgcgcttgaattattattattcagGAATGGCGGTTGCACGAGATATCAAGG
GGAAGCGGGAACGTGTAGTTGCTGTCATCAGTAATGAAACAACAATGGCAGGTCAGATTT
ACGAGGCAATGAGTAATGCTGGATATTTGGATTCGAACATGGTTGTGATATTAAACGACA
GCCGCCATTCAATAAACCCAAAACTTGAGGAGGCCACCAAAACCCCAATAAACGCACTTT
CCAGTACTTTAAGCAAGCTGCAGTCAAGTAAAATCTTCAGGAAATTCAGAGAAGTAGCTA
AGgtatgattttcgatgatttttcatgtattattcgtctagatttaatctaataggatgt
gtttgggtgaatgattttgggtgaggattggatttttgataaggttcgtaagttgaaatt
gggttaaccacactttgcagtttgcacccttatggttagatagaattgcaattcacgctc
ctgtgctattttcgatcaattcttgtagcgccatcttttttttttgtttttaaattttt
cttgtttcagGGATTTACTAAAAGAATCGGTAAAGGCATGCATGAGTGGGCGGCCAAAAT
CGACGAATATGCACGTGgtatgatcggtccgcttggatcaactctctttgaagaactcgg
attgtattacattggccctgtggatggacacaatatcgaagaccttgtatgtgttcttcg
tgaagtggcgtccttggattcaatgggccctgttttggtacatgtcataacaaaggaaaa
tcaggaagtagtagaagaagaagatcaaacaattaagatattaaatcaaaatttgaaagg
tttgttttttctcttggggtttttgacatttcgtagccttgaagtttgaccaaaatcgca
ctttggtccccctgctttcaattagaacatatagcaccttaatgtttcaaatttattcca
ctacagtccaagtcaccatccccatcacaattttggatagaaaacagtcacctgccgcgc
acttcattgtttttaaggggctttatgagcattcccaacctcttcctttctcttctccca
ttctccttcatgcccaaatactcataaatatatatatatacacatatagatacacataaa
tgtttttttttagaagggcaaaggaagaggatagaaatgcccataagttccccttaaaaat

agttgggtgcacgataggtgactgtttactgtccaaaattgtaacgttgctatagggttg
gactgaagtgggatgattacgaaacttcaagatgtcatgtgttcaaattgaaacaatggg
gaccaaagtgaaataatggtaaaacttcaggggtgcaaaatgtcaaaaccccttttttct
tcatctgtttgatctcttcaaaacataaatgcaaagagagagtttttgatatcttagttc
tttctcatgtttcattgtttagggcagATTCATTATCAAATGATGGTCGGCCTCAAACGT
ATAGCGATTGTTTTGTCGAGTCATTGGTTATGGAGGCTGAAAGAGACAAAGATATCGTGG
TAGTTCATGCAGGAATGGGAATGGAACCGTCTCTTAAAATGTTTGAGAAGCGGTTTCCAG
ACCGGTTTTTTGATGTGGGGATGGCTGAGCAGCATGCTGTGACATTCTCTGCCGGTTTGT
CGTGTGGAGGACTGAAGCCGTTCTGTGTAATCCCGTCTTCCTTTCTTCAGAGAGCTCTTG
ATCAGgtctctctctagattgtgtatataagctctctttctctctaaaatggcttcggaa
catatttaatgactaggtggagaaaactaacacactgtttggtaacgctttctcaagtat
tttttagggttaattgccttttaccgtcgtctacttcaacaacctacactttgccccctt
ctactaataataaattagaaaacgaacttttattttttgttttgctaaacggcatatgtt
aaaaaaatgagtgaaattaaaatttccatctaaatttcatatgaaatggacaaaacaccc
ctttattttgtttttttattttattgaaaaaaagttaaagaaaaatataaatagagaggga
aagtgagctagagagagaaaataccggtttttcacttttattggctttaaatattattta
aattaaaacaataacgggcaattaagtcatttgtattgtttttgaataagttgtgttttg
ctacctttagtcaagagtagaagggggtgatgtgtagttttttaaagtagaggggttaaa
atgtaacaggaccaaagtattaggggttaaaacgcaattaaccctatttttttatgataaa
aaaaagttaatctattataaaagtatttttatttaaaagtaaaataaaaaatcaaaagta
tatttgacagtaaagagagaccatcttgcttttatctctatatagtatctcttcatagcc
gggagaatcaaaagttaaatctgttaaaaaaatatttttacttcaaaaataaaagtaaaa
tcgaaagtatttctgaactggataaagagaagggtttttttggtgaatacttcaagaaatt
aaaattttagctcatttagtccacacgctttagtaatttttcaaaaagtccaaagttact
gtaatatataaaagttattgtaaatttttcatgtaaaaaattgcaataatttttctgacg
tacaatgacttttgaatttttgccagtaaatttggatttctgaaaaaattactgtagttc
gtggactgcatgatttaaaattttaatttcgtgaactgttaaccaaaaaaccctaaagag
aatatattggttttatcccctctgccaatctattaaaatataaaattcaagaaataggtt
ttttttttaattaaaacaaaacaaagaaagaaaaagaaaaagaaaaaacttaaaatcac
gttttgtactttttttatttttttatttttttcagtgcaaaacacacactactttccaact
aaaaaagtacttcaaaaaacgttaccaaacggtgcctaaatttacgtttttctgattttt
ttctactttccttgtgtcatcgttagGTAGTTAATGACGTAGATCGACAAAAGATTGCGG
TGCGTTTTGTCATTACGAGCGCAGGGTTGGTTGGATCAGATGGATCAACCAACTGTGGGG
CATTTGACATAACATTCATGTCTTGTTTGCCAAACATGATTGTGATGGCGCCTTCCGATG
AGGCTGAGCTCTCTCACATGGTGGCCACAGCTGCCCATGTCGATGATAGGCCCGTTTGCT
TCCGTTATCCTAGAGGAGCGGTTGCTGAGATCAACTTAATGACCGCCGGAGTCCCCATTG
AGgtaatgactatggaacttcttgctagtctagtatttttttttcttcccccctaatagttc
catgccctaatgggtacctaaggtggtgttcgtttaggggttgtttggttaagaactcga
gtacgtattctcaagtcctgtttggttacagttttcgttccttgatttctgttcctgctc
tcaaaattttgggattaacgtacagaaacgtgtttggtaaacacctttctgttccaaaaa
atttgggaatatttcatgaattccttaagaatctaagaaacaaaaaaaaaaatgttgattc
ctattcactcccacaaaaccattctccctaccaccccatcggccaccatctcctcccggc
caccaccaccataccggctatcaccaccctgtcggccaccaccaccactacaaccaccat
atttttaaagaaaaaaaagaacaaatgttgctaccaaaattgtttcttgtttttgttctc

ataaattaaaagaaacaattttaggacaatggtcgttattaaacatgtttctattcccgg
aatactccagaccagaaatagaattcccatcccagtttattggaagtaggaacagaaaca
ctaaccaaaagccagttacgtcaccttagtgcttacattttttttgtttcttttcccag
aaaatatagaacagaaacagaaatctgggagaaaaacaatctatttttgttgcatccccc
aaaatctgttccaaaaatcttggaaaaataaaaacaaaaacactaaggtcgtgtttggtt
gcatggaattagagccttgaaattggaattagaaatgaactagaaatgaacgcatataaa
aattaaaaaattcacatgtattcatttcgaattccaattccatgccttcaattccgagct
cccaaacactacctaaacttccgttctaaatatatatactcatttcccgaacccaataat
catatatgaaccagATTGGCAAAGGGAGAGTCCTTATAGAAGGTAAAGACGTTGCTTTGC
TTGGCTACGGCGCCATGGTACAAAACTGTGTAAGAGCTCAATCCATTCTCTCAAACCTCG
GCGTGGAGGTGACCGTTGCCGATGCGAGATTCTGCAAGCCGCTCGACGTCAGTCTCGTCA
GGAAACTATGCGAAGAACACGCCTTCTTGATCACGGTCGAGGAAGGGTCGCTTGGAGGGT
TTGGGTCCCACGTTGCTCAATTCCTTTCACTTGATGGCCAACTCGACGGGAAGGTCAAGg
tataatcgagatcacctaagttctttagtagtttaatttttttgatgaataaatttatgg
aacgggtgtgtgtatatagcaaaaaagggtgtatgtttagcagctctatgcagtcaaaag
ttgcgcaaaattacaaatatgcccaacatcgagataaatagtagagaaaatcggtgaaaa
gtttatttttttaaatctaattggtgaaaagtccttttgtcttattaggttttgatttag
tcctttaagttagattttacttgtgttttaatcccacattgcaggaattgtagggatatt
gcagggactgtaaggagtcttttagtgagtacatttggtttttatagtgaattctattac
atgtaactaatgttagttacaatttcataattgttacttttcacgcatattgggtatttt
tagttacatgtacttatataatattagttacatgttacaattgcatatatatgtataata
ttttgtgacgtaaccgaaaaaatactaaactttttagttacatattagagacatgtgact
aaaaggttacatattattcaaacatgtaactgaaaaatattaaactttttagttacatat
tattggaacatgtaattggaaaaaaaaaaaaaaaaaagtagttacctgtaactagtacag
taatattacacacgaagtagttacatgtaactggcgcatggttatcacgtgaacaaggct
tccaccttttaaaacagatgggactaaatcaaaagtttttaccgcgtgtaggacttttc
atcaattagagtctaaatggaagacctcccaccgattgtccctaaatagtaattggcaat
agcctcttttactataggggtgtttgtttagcgaaaaaaggtgtacatttagaaaccacg
cagagtcaaaagttgcatgaaattacaaaaatgcccttacattgagatcaattgtaattg
cgatagcttctctcccatagaagaagggggttatgtttagcaaaaaagggtgtacattta
gaagcggtgtggagtcaaaagttgcataaaattaccaaaatgcccgacattcagatcact
tgtaattggctatagcttcttttactgtagaaggggtgtatgtatagcaaaaaaaggtgt
acatttagaaataacgcggagtcaaaattgtaataagtaaaaatttgtctaagtttcata
tccaataatttatttatttattttttaattttttttaacagTGGAGACCGATTGTTCTAC
CAGACAACTACATTGAGCACGCATCGCCGAAGGAGCAACTTGCTCTTGCAGGGTTGAGCG
GACATCACATTGCAGCAACGGCGTTAAGCCTTCTTGGTCGCACTCGTGAAGCCTTACTCT
TGATGTGCTAA
>*EuDXS4* EU0108841.1 CAFEUEU0108841 Chr05:1823243-1831299(-)
ATGGGTGCTGCTTCTCCTCAGTACCCATTTGGTTTCGCCTCCAATTTCCATGGAAATTCG
AGGGTGTTTTCTCCCAAAGTGCAGTTCTCAGCTGTTAATTTCTCTTCCGGGGGGCAATTT
TCGCGAAGGGATTCCGGCCAAATTTCAGCCTCCATACTTAATTTCAAGgtacgatgcttt
gattttccttaggtagcgtttgggagaacgaaattgaaaccatttggaattggaatttga
aatgaacgcatactatcaattcaaattccaattccatgcaaccaaacactaccttatagt
tttccccctttatagaaattgttagattcctgtcccaatatcgatcacttgagagttctc

ggttaccaccatatggttttaaaaacaactggtataagaggcaaggttataagaatttgc
ctcacatttgtacattttctaatgatcaaatcatccgttcaagttggggttgcaaattgt
tcatcattctttcgtaatcaaatcattcgtacaaacttggggttacattgaatggcggat
gctagcttcctctcccacatcgagttcccaaactactatataaatcaaaagagctactcc
aattgacactatatggttttagattggaacctcttaacataaattaagaattgtattgag
ggtaaactttgatatttactttctctacaaaatctaaatcaacaattgaaatggctctcc
gactgtccaattgtaaaaaatatttaaaatataaaatattcctgatttattgtatagata
atatagattttaatccaatacaataattttgaacgtcttatagttagttcaaatacatta
atcatgaaaatacattttgttcgagagattgatatttggtttctcaacaaaatctaaatc
aacagttgcaaatgtctccaaccatccaatggtaaaaaatctctgaaatgtcgaatatgt
cttgatttattatatagattatacatattttaattcaatacattaacctcagttagtgct
tttcttttgcaactcctgatgtagGAACGCTTTGGTATACATTCTCTACCTGATGTTGGC
GATGTATTTAGGGAGAATGCGCCAACACCCATGCTTGATACCATCGAAAATCCCATGCAT
TTGAAGAATTTGTCTCCAAAGgttaagaaatcggcgttctttatttacatatttttgttg
ttttaagcgcgaggcttttattttaaatccgatactaagttgattgattgtgtgcagGAA
CTAAAACAATTAGCGAATGACATCAGATCGGAATTATACCATACATTGTCAAAAATACAA
AAACCGTTCAAAGCTAGTTTGGCAGCGGTGGAGCTGACGATTGCAATACATCATgttttt
caagctccattggacaagatattgtgggacatgggggaacaagtacatatatatgtgtat
atatatacatacatataattatatatgtttatttattataaattaggacggtgtgggctt
catataataattggtgtcaatagaatggatgagatctgttagtgaaaatgtggatttaat
ctcaaccattcaactgaagaaatattttcatcctttggaccatatatttgaagagattct
agtctttctatcgaatataattactttgtagagcacacaccattctaatttataatacaa
aatttattatttttggaatttgttagttgaaatttgaaattacataatttttttattga
aataatattaatgcgtgcctttaggcacaagcacttactagtgtgtgtatatatattttg
tctttgatgtatgcagGCATATGCGCATAAGATCCTCACTGGGAGACGGCCCCTCATGGA
TACAATTGGACAAACGAATGGTCTTTCGGGTTACACATCCCGTTTTGAGAGTGAATTTGA
TCCATTTGGTGCAGCGCATGGATGCAGTAGTGTTTCTGCTGGACTTGgtatgtgtgtgtg
tatatatacaacaacaacaacaaccaaggctctgtcccactacaatatatatatgtgtgt
gtgtatacatatatattggtgtttttttttaaatttttaacaagtgtgttttgaaatagt
ctaaagtctaatccaatctgctttgtttggctgtgttttatagatattcaatgatgcctc
caatttcaattctcgacaaaaaacaaaaagagggttaatggcactttacctcgttgtggt
ttgaccgaattgcacttatccccccttggtattttcgtaagcacttacccatctgtggttc
agattaaagtgcaatggtcacattttccattgaagttaatggtcaaacctcaaacgtcaa
aattaataaattatttaatatcaattgacatttataccaccagatcgggtcgagtaggat
aatatccatttgccttctgtttttttcaccaggtaggatcgggttcaatataagggtaaa
ttttcaatgtcacgcaattctattagttatctaaccgttgcactttagtttggaccatgg
ggggtaagtgcttatggatatactacagggaggtaaaatgcaatttggtcaaaccagggg
taaaatgcgttaaccccaaaactaaataaatcattaacagctccacctctatgctatttg
agtatgaaaacttaaaattactaaataaatcattaacagctccacctctatgctatttga
gtataaaaacttcaaattttgagcatgatgcgcttgaattattattattcagGAATGGCG
GTTGCACGAGATATCAAGGGGAAGCGGGAACGTGTAGTTGCTGTCATCAGCAATGAAACA
ACAATGGCAGGTCAGATTTACGAGGCAATGAGTAATGCTGGATATTTGGATTCGAACATG
GTTGTGATATTAAACGACAGCCGCCATTCAATAAACCCAAAACTTGAGGAGGCCACCAAA
ACCCCAATAAACGCTCTTTCCAGTACTTTAAGCAAGCTGCAGTCAAGTAAAATCTTCAGG

AAATTCAGAGAAGTAGCTAAGgtatgattttcgatgatttttcatgtattattcgtctag
atttaatctaataggatgtgtttgggtgaatgattttgggcgtggattggattttcaata
aggttcataagttaaaattgggttaacgacactttgcagtttgcacccttatggttagat
agaattgcaattcacgctcctgtgctattttcgatcaattcttgtagcgccatctttttt
ttttttttaatttttcttgtttcagGGATTTACTAAAAGAATCGGTAAAGGCATGCATGA
GTGGGCGGCCAAAATCGACGAATATGCACGTGgtatgatcggtccgcttggatcaactct
ctttgaagaactcggattgtattacattggccctgtggatggacacaatatcgaagacct
tgtatgtgttcttcgtgaagtggcgtccttggattcaatgggccctgttttggtacatgt
cataacaaaggaaaatcaggaagtagtagaagaagaagatcaaacaattaagatattaaa
tcaaaatttgaaaggtttgtttttctcttgggggtttttgacatttcgtagccttgaagt
ttgaccaaaatcgcactttggtccccctgctttcaattagaacatatagcaccttaatgt
ttcaaatttattccactacagtccaagtcaccatccccatcacaattttggatagaaaac
agtcacctgccgcgcacttcattgtttttaaggggctttatgagcattcccaacctcttc
ctttctcttctcccattctccttcatgcccaaatactcataaatatatatatatacacat
atagatacacataaatgtttttttttagaagggcaaaggaagaggatagaaatgcccataa
gttccccttaaaaatagttgggtgcacgataggtgactgtttactgtccaaaattgtaac
gttgctatagggttggactgaagtgggatgattacgaaacttcaagatgtcatgtgttca
aattgaaacaatggggaccaaagtgaaataatggtaaaacttcaggggtgcaaaatgtca
aaacccctttttcttcatctgtttgatctcttcaaaacataaatgcaaagagagagttt
ttgatatcttagttctttctcatgtttcattgtttagggcagATTCATTATCAAATGATG
GTCGGCCTCAAACGTATAGCGATTGTTTTGTCGAGTCATTGGTTATGGAGGCTGAAAGAG
ACAAAGATATCGTGGTAGTTCATGCAGGAATGGGAATGGAACCGTCTCTTAAAATGTTTG
AGAAGCGGTTTCCAGACCGGTTTTTTGATGTGGGGATGGCTGAGCAGCATGCTGTGACAT
TCTCTGCCGGTTTGTCGTGTGGAGGACTGAAGCCGTTCTGTGTAATCCCGTCTTCCTTTC
TTCAGAGAGCTCTTGATCAGgtctctctctagattgtgtatataagctctctttctctct
aaaatggcttcggaacatatttaatgactaggtggagaaaactaacacactgtttggtaa
cgctttctcaagtattttttagggttaattgccttttaccgtcgtctacttcaacaacct
acactttgccccccttctactaataataaattagaaaacgaacttttattttttgttttgc
taaacggcatatgttaaaaaaaatgagtgaaattaaaatttccatctaaatttcatatgaa
atggacaaaacaccccctttattttgtttttttattttattgaaaaaaaagttaaagaaaaat
ataaatagagagggaaagtgagctagagagagaaaataccagtttttcacttttattggc
tttaaatattatttaaattaaaacaataacgggcaattaagtcatttgtattgtttttga
ataagttgtgttttgctacctttagtcaagagtagaagggggtgatgtgtagttttttaa
agtagaggggttaaaatgtaacaggaccaaagtattaggggttaaaacgcaattaaccct
attttttatgataaaaaaaaagttaatctattataaaagtatttttatttaaaagtaaaat
aaaaaatcaaaagtatatttgacagtaaagagagaccatcttgcttttatctctatatag
tttctcttcatagccgggagaatcaaaagttaaatctgttaaaaaaatatttttacttca
aaaataaaagtaaaatcgaaagtatttctgaactggataaagagaagggtttttttggtga
atacttcaagaaattaaaattttagctcatttagtccacacgctttagtaatttttcaaa
aagtccaaagttactgtaatatataaaagttattgtaaatttttcatgtaaaaaattgca
ataatttttctgacgtacaatgacttttgaatttttgccagtaaatttggatttctgaaa
aaattactgtagttcgtggactgcatgatttaaaattttaatttcgtgaactgttaacca
aaaaaccctaaagagaatatattggttttatcccctctgccaatctattaaaatataaaa
ttcaagaaataggttttttttttttaattaaaacaaaacaaagaaagaaaaagaaaaaga

aaaaacttaaaatcacgttttgtactttttttattttttattttttcagtgcaaaacac
acactactttccaactaaaaaagtacttcaaaaaacgttaccaaacggtgcctaaattta
cgtttttctgatttttttctactttccttgtgtcatcgttagGTAGTTAATGACGTAGAT
CGACAAAAGATTGCGGTGCGTTTTGTCATTACGAGCGCAGGGTTGGTTGGATCAGATGGA
TCAACCAACTGTGGGGCATTTGACATAACATTCATGTCTTGTTTGCCAAACATGATTGTG
ATGGCGCCTTCCGATGAGGCTGAGCTCTCTCACATGGTGGCCACAGCTGCCCATGTCGAT
GATAGGCCCGTTTGCTTCCGTTATCCTAGAGGAGCGGTTGCTGAGATCAACTTAATGACC
GCCGGAGTCCCCATTGAGgtaatgactatggaacttcttgctagtctagtatttttttc
ttccccctaatagttccatgccctaatgggtacctaaggtggtgttcgtttaggggttgt
ttggttaagaactcgagtacgtattctcaagtcctgtttggttacagtttttgttccttg
atttctgttcctgctctcaaaattttgggattaacgtacagaaacgtgtttggtaaacac
ctttctgttccaaaaaatttgggaatatttcatgaattccttaagaatctaagaaacaaa
aaaaaaatgttgattcctattcactcccacaaaaccattctccctaccaccccatcggcc
accatctcctcccggccaccaccaccataccggctatcaccaccctgtcggccaccacca
ccactacaaccaccatatttttaaagaaaaaaaagaacaaatgttgctaccaaaattgtt
tcttgtttttgttctcataaattaaaagaaacaattttaggacaatggtcgttactaaac
atgtttctattcccggaatactccagaccagaaatagaattcccatcccagtttattgga
agtaggaacagaaacactaaccaaaagccagttacgtcaccttagtgcttacatttttt
ttgtttcttttcccagaaaatatagaacagaaacagaaatctgggagaaaaacaatctat
ttttgttgcatcccccaaaatctgttccaaaaatcttggaaaaataaaaacaaaaacact
aaggtcgtgtttggttgcatggaattagagccttgaaattggaattagaaatgaactaga
aatgaacgcatataaaaattaaaaaattcacatgtattcatttcgaattccaattccatg
ccttcaattccgagctcccaaacactacctaaacttccgttctaaatatatatactcatt
tcccgaacccaataatcatatatgaaccagATTGGCAAAGGGAGAGTCCTTATAGAAGGT
AAAGACGTTGCTTTGCTTGGCTACGGCGCCATGGTACAAAACTGTGTAAGAGCTCAATCC
ATTCTTTCAAACCTCGGCGTGGAGGTGACCGTTGCTGATGCGAGATTCTGCAAGCCGCTC
GACGTCGGTCTCGTCAGGAAATTATGCGAAGAACACGCCTTCTTGATCACGGTCGAGGAA
GGGTCGCTTGGAGGGTTTGGGTCCCACGTTGCTCAATTCCTTTCACTTGATGGCCAACTC
GACGGGAAGGTCAAGgtataatcgagatcacctaagttctttagtagtttaattttttg
atgaataaatttatggaacgggtgtgtgtatagcaaaaaagggtgtacatttagcagctc
tatgcagtcaaaagttgcgcaaaattacaaatatgcccaacatcgagataaatagtaatt
ggcaatagcctcttttactataggggtgtttgtttagcaaaaaaaggtgtacatttagaa
accacgcggagtcaaaagttgcatgaaattacaaaaatgccctgacattgagatcaattg
taattgcgatagcttctctcccatagaagaagggggttatgtttagcaaaaaagggtgta
catttagaagcggtgtggagtcaaaagttgcataaaattaccaaaatgcccgacattcag
atcactttgtaattggctatagcttcttttactgtagaaggggtgtgtgtatagcaaaaa
aaagtgtacatttagaaatcatgcggagtcaaaattgtaataagtaaaaatttgtctaag
tttcatatccaataatttatttatttatttttatttttttaacagTGGAGACCGATTG
TTCTACCAGACAACTACATTGAGCACGCATCGCCGAAGGAGCAACTTGCTCTTGCAGGGT
TGAGCGGACATCACATTGCAGCAACGGCGTTAAGCCTTCTTGGTCGCACTCGTGAAGCCT
TACTCTTGATGTGCTAA
